# Allele-specific SHAPE-MaP assessment of the effects of somatic variation and protein binding on mRNA structure

**DOI:** 10.1261/rna.064469.117

**Published:** 2018-04

**Authors:** Lela Lackey, Aaztli Coria, Chanin Woods, Evonne McArthur, Alain Laederach

**Affiliations:** 1 Department of Biology, University of North Carolina, Chapel Hill, North Carolina 27599, USA; 2 School of Medicine, Vanderbilt University, Nashville, Tennessee 37232, USA

**Keywords:** RNA secondary structure, SHAPE-MaP, *TPT1*, *LCP1*, riboSNitch, somatic mutation

## Abstract

The impact of inherited and somatic mutations on messenger RNA (mRNA) structure remains poorly understood. Recent technological advances that leverage next-generation sequencing to obtain experimental structure data, such as SHAPE-MaP, can reveal structural effects of mutations, especially when these data are incorporated into structure modeling. Here, we analyze the ability of SHAPE-MaP to detect the relatively subtle structural changes caused by single-nucleotide mutations. We find that allele-specific sorting greatly improved our detection ability. Thus, we used SHAPE-MaP with a novel combination of clone-free robotic mutagenesis and allele-specific sorting to perform a rapid, comprehensive survey of noncoding somatic and inherited riboSNitches in two cancer-associated mRNAs, *TPT1* and *LCP1*. Using rigorous thermodynamic modeling of the Boltzmann suboptimal ensemble, we identified a subset of mutations that change *TPT1* and *LCP1* RNA structure, with approximately 14% of all variants identified as riboSNitches. To confirm that these in vitro structures were biologically relevant, we tested how dependent *TPT1* and *LCP1* mRNA structures were on their environments. We performed SHAPE-MaP on *TPT1* and *LCP1* mRNAs in the presence or absence of cellular proteins and found that both mRNAs have similar overall folds in all conditions. RiboSNitches identified within these mRNAs in vitro likely exist under biological conditions. Overall, these data reveal a robust mRNA structural landscape where differences in environmental conditions and most sequence variants do not significantly alter RNA structural ensembles. Finally, predicting riboSNitches in mRNAs from sequence alone remains particularly challenging; these data will provide the community with benchmarks for further algorithmic development.

## INTRODUCTION

RiboSnitches are single-nucleotide variants (SNVs) that cause changes in RNA secondary structure (Halvorsen et al. 2010; Wan et al. 2014; Gotea et al. 2015; Kutchko et al. 2015; Lu et al. 2015; Solem et al. 2015). These riboSNitches result in RNAs with different structures, leading to potentially different regulatory and functional abilities. Approximately 15% of inherited SNVs change RNA structure in family trio transcriptome-wide structural experiments (Wan et al. 2014). However, in general the function of structure in messenger RNAs (mRNAs) is unclear (Bartel 2009; Scharff et al. 2011; Dethoff et al. 2012; Li et al. 2014). Certain specific structures, such as the Iron Responsive Element and the Histone Stem–Loop, play central roles in post-transcriptional regulation of the mRNAs in which they occur (Gallie et al. 1996; Marzluff et al. 2008; Ma et al. 2012). For instance, the Iron Response Element within *FTL* mRNA normally forms a hairpin structure and is regulated by an iron response element binding protein (Burdon et al. 2007). Several inherited SNVs within *FTL* alter the structure of this element and are associated with hyperferritinemia cataract syndrome (Halvorsen et al. 2010; Martin et al. 2012).

The coupling of next-generation sequencing with chemical and enzymatic probing, such as in SHAPE (Selective 2′ Hydroxyl Acylation by Primer Extension) methodology (Siegfried et al. 2014; Lu and Chang 2016; Zubradt et al. 2017) enables structure analysis at unprecedented scale, including transcriptome-wide RNA secondary structure determination (Kertesz et al. 2010; Underwood et al. 2010; Zheng et al. 2010; Lucks et al. 2011; Ding et al. 2014; Incarnato et al. 2014; Rouskin et al. 2014; Wan et al. 2014; Del Campo et al. 2015). However, the final structures and even existence of RNA secondary structure under biological conditions remains controversial as secondary structure probing within cells yields significant variability depending on the approach (Ding et al. 2014; Rouskin et al. 2014; Spitale et al. 2015; Watters et al. 2016a; Lee et al. 2017). Although some well-studied small RNAs, such as the hairpin ribozyme and bacterial RNase-P, fold to near native conformations in the absence of cellular components (Donahue et al. 2000; Lindell et al. 2005), the specific effects of the cellular environment on RNA structure remain poorly understood (Schroeder et al. 2004; Mahen et al. 2010; Zemora and Waldsich 2010; Kubota et al. 2015; Smola et al. 2015a, 2016). We know that during translation ribosomes assisted by helicases must unfold both the mRNA coding region and, likely, structured elements in the untranslated regions (UTRs). However, it is a thermodynamic reality that, when not actively unfolded, single-stranded RNA will rapidly form intramolecular base pairs (Das et al. 2003). Thus, we expect that different subpopulations of mRNAs exist in the cellular environment, and, if we probe their structures in bulk, we will observe signals averaged over the ensemble of subpopulations, including structures specific to cellular and in vitro conditions.

We chose to look in-depth at two specific mRNAs, *TPT1* and *LCP1*. The first mRNA, *TPT1* (*tumor protein, translationally controlled 1*), was originally characterized as a sequestered mRNA that is translationally induced during growth conditions (Gross et al. 1989). We are particularly interested in *TPT1* because it is thought to have extensive secondary structure, even to the extent of activating the double-strand RNA recognition protein PKR (Bommer et al. 2002; Nussbaum et al. 2002). Although not known to be as structured as *TPT1*, *LCP1* (*lymphocyte cytosolic protein 1*) is also over-expressed in many different cancers and may be involved in cell mobility (Shinomiya 2012; Van Audenhove et al. 2016).

We develop here a combined experimental and computational method to confidently detect riboSNitches arising from inherited SNVs as well as from somatic mutations identified in cancers. We take advantage of recent chemical probing techniques that allow us to rapidly obtain high-resolution structural information on full-length transcripts (Siegfried et al. 2014; Smola et al. 2015b), while also facilitating a rapid, allele-specific sorting of reads for rapid and accurate riboSNitch detection. We compare the ability of traditional SHAPE-MaP to detect riboSNitches and find that using allele-specific sorting decreases the background noise and improves riboSNitch detection. Combined with a thermodynamically rigorous framework that enables us to use experimental SHAPE data as pseudo-free energies in nearest neighbor RNA folding free energy models, we are able to directly visualize the Boltzmann suboptimal ensemble and how these variants affect the ensemble (Deigan et al. 2009; Hajdin et al. 2013; Woods et al. 2017). Finally, to verify that in vitro determined riboSNitches are biologically relevant, we test how stable the structures are within *TPT1* and *LCP1* mRNAs by performing SHAPE-MaP in the presence and absence of cellular proteins. With these techniques, we determined that *TPT1* and *LCP1* mRNAs are structurally robust with high correlations between protein-bound and -unbound experiments. We also identified five riboSNitches, including two arising from somatic mutations within the coding sequencing of *LCP1* and three within the UTRs of *TPT1*.

## RESULTS

### Variation and conservation within *TPT1* and *LCP1* mRNAs

**FIGURE 1.**
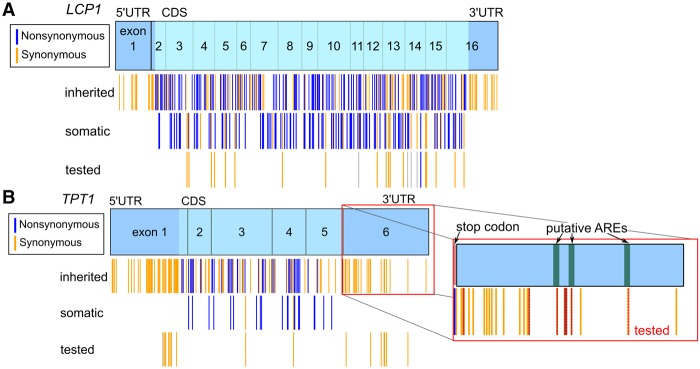
Variation within *LCP1* and *TPT1*. (*A*) *LCP1* mRNA is long (coding sequence of 1884 nt) and is composed of 16 exons. (*B*) *TPT1* has three isoforms and multiple alternative polyadenylation sites, of which NM_003295 with an early polyadenylation site is the most prevalent (∼1000 nt in length). All identified inherited SNVs and somatic mutations within these genes are shown (blue, nonsynonymous; gold, synonymous). We tested all synonymous somatic mutations within the *LCP1* and *TPT1* CDS regions and selected inherited variants.

Like many other human genes, *LCP1* and *TPT1* harbor somatic mutations from various cancers and have extensive inherited variation (Fig. 1A,B; Sherry et al. 2001; Forbes et al. 2015). We are particularly interested in the potential effects of these mutations on RNA structure. We performed in vitro SHAPE-MaP experiments on a subset of somatic and inherited SNVs. Our goal was to screen a broad range of genetic and somatic mutations to assess potential consequences on RNA structure. We obtained somatic mutations from the Catalogue of Somatic Mutations in Cancer (COSMIC) database, which contains primarily exome sequencing from a variety of different cancers, and we obtained inherited polymorphisms from the National Center for Biotechnology Information (dbSNP) database. We focused our experimental analysis on synonymous somatic mutations in the coding sequence because these mutations will not affect the protein product and are therefore more likely to be functional riboSNitches (Shabalina et al. 2013; Hunt et al. 2014; Supek et al. 2014; Gotea et al. 2015). *LCP1* has only one isoform and a long coding region with 21 synonymous somatic mutations (Fig. 1A). In the most commonly expressed isoform of *TPT1* (NM_003295), there are only two synonymous mutations in the coding sequence (Fig. 1B). We therefore expanded our experimental investigation into a subset of inherited SNVs from the 5′ and 3′ UTRs, concentrating on putative functional regions such as predicted AU-rich elements (AREs) (Fig. 1B). Our selected subsets of somatic and inherited variants did not have different conservation scores from randomly selected nucleotides (nt) as a group in either *LCP1* or *TPT1*.

### Allele-specific sorting greatly improves riboSNitch detection

**FIGURE 2.**
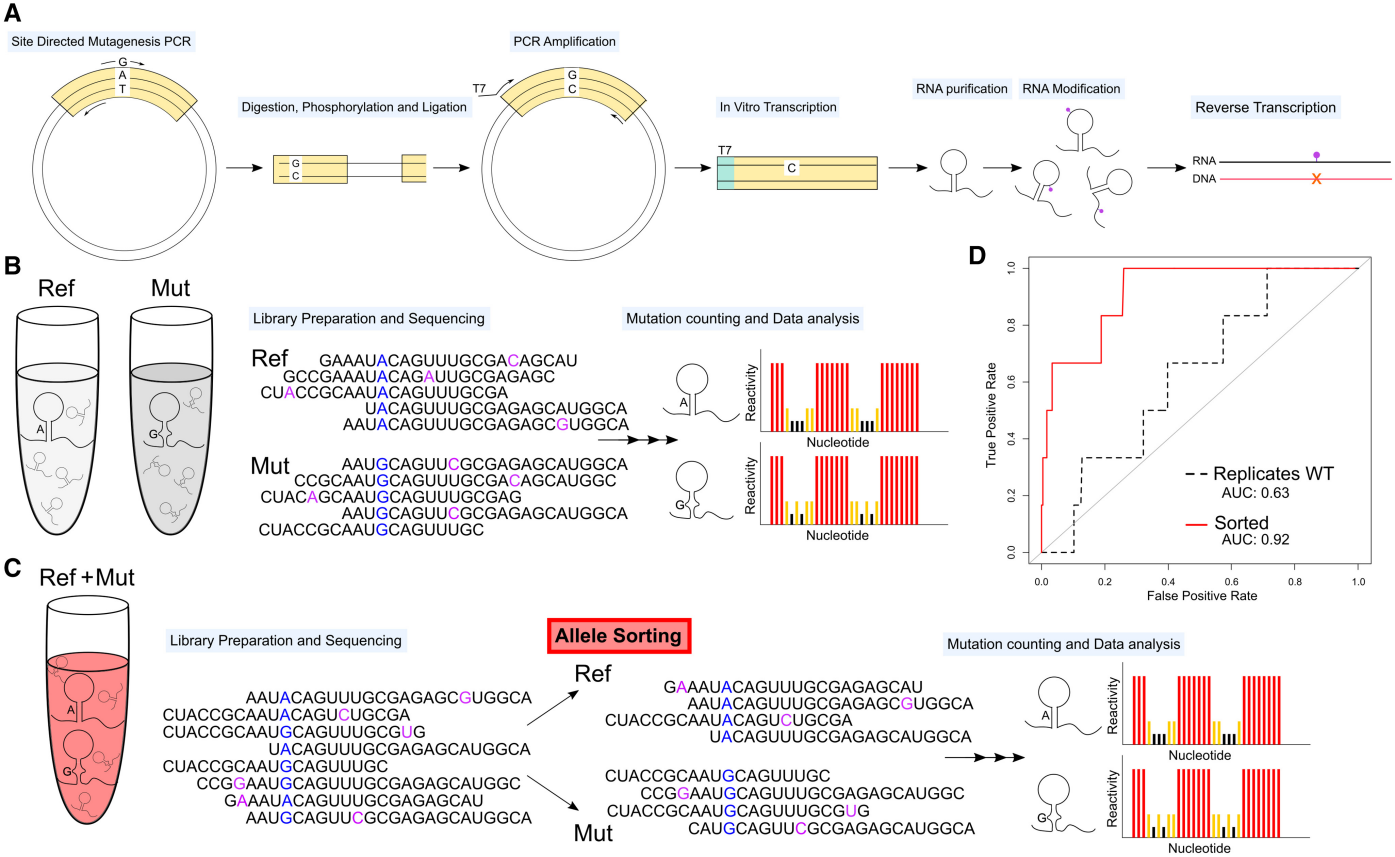
High-throughput strategy to identify riboSNitches is improved by clone-free allele-specific sorting. (*A*) We performed site-directed mutagenesis followed by SHAPE-MaP. (*B*) In a traditional experiment, reference and mutant RNAs are modified and prepared for sequencing in separate tubes. (*C*) In our novel protocol, reference and mutant simultaneously undergo modification. The sequencing reads are sorted based on the reference/mutant allele and SHAPE reactivities are calculated from the sorted reads. (*D*) By simulating the traditional experimental approach, we have poor differentiation (AUC 0.63, dotted line), while simulating our novel within sample approach results in a dramatic improvement (AUC 0.92, red line).

We were able to obtain SHAPE data on 37 selected variants within *LCP1* and *TPT1* using a novel, high-throughput SHAPE-MaP protocol (Siegfried et al. 2014; Smola et al. 2015b). Briefly, we used a clone-free, site-directed mutagenesis technique to create selected variants, transcribed the RNA variants, probed the RNA with 1M7 or the negative control, DMSO, and proceeded with error-prone reverse transcription to fix the adduct locations as mutations in the cDNA (Fig. 2A,C). Since the mutagenesis step (Fig. 2A) is not 100% efficient, a percentage of wild-type (WT) sequence remains in the amplification, and WT RNA is spiked into the reaction, resulting in both alleles being simultaneously probed. This is in contrast to a strategy in which both alleles are probed in separate tubes, effectively introducing replicate variability into their SHAPE signals (Fig. 2B). The novel experimental protocol used here takes advantage of SHAPE-MaP's read-through reverse transcriptase step and allows allele-specific sorting of reads (Fig. 2C). Therefore, our strategy for obtaining matched SHAPE data for both alleles uses an in vitro mutagenesis approach, enabling us to solve the structures of each allele by sorting reads prior to generating SHAPE data. This approach has the advantage of being internally consistent, i.e., each allele is probed under identical conditions, increasing the sensitivity of the assay.

To evaluate the improvement resulting from modification of both alleles in the same tube we estimated the false discovery rate of riboSNitches in replicate WT SHAPE data. We compared each WT SHAPE data set to the total average WT SHAPE. We expect that replicates of the same RNA sequence should have no riboSNitches and that any differences in the data are due to replicate noise and are therefore false positives (only manually confirmed nonchanger samples were used to create the average total WT SHAPE in these comparisons). We used the classSNitch algorithm, which establishes if two SHAPE traces are different using a random forest classification algorithm (Woods and Laederach 2017). ClassSNitch classified 52% of these WT to WT comparisons as riboSNitches, suggesting a false discovery rate of a traditional nonallele-sorted experiment at 0.52.

We also generated within-sample data sets that were ratiometrically normalized to match our allele specific sorting to control for variations in sequencing depth, which are known to impact signal to noise in SHAPE-MaP data (Siegfried et al. 2014). In this case, we expect that within-sample data sets should have no riboSNitches and that any differences in the data are therefore false positives. We used the classSNitch algorithm (Woods and Laederach 2017) and found that within sample comparison dramatically lowered the false positive rate, with only 9% of samples falsely identified as a riboSNitch. We performed receiver operator curve (ROC) analysis to control for classSNitch sensitivity on the ratiometrically sorted (red line, Fig. 2D), and replicate WT (black dotted line, Fig. 2D) data sets. The result is a remarkable improvement in riboSNitch detection accuracy (area under the curve, AUC) increase from 0.63 to 0.92.

Adding allele-specific sorting to the SHAPE-MaP procedure greatly improves the detection of single-nucleotide mutations that change RNA structure. This technique is not without cost, however, as only reads that span the mutation site or are paired with a read spanning the mutation site can be accurately sorted. This restricts SHAPE data to within a few hundred nucleotides of the mutation. The vast majority of base pairs even in highly structured RNAs like the Ribosome tend to be short-range; thus it is reasonable to expect riboSNitches to be local and allele-specific sorting adequate for detection of the majority of structure changes (Doshi et al. 2004; Kladwang et al. 2011a,b; Cordero et al. 2012; Woods and Laederach 2017). In addition, improvements in sequencing technology readily increase read lengths, and as data are collected with longer reads, allele-specific sorting will allow the detection of larger structural changes.

### Identification of five riboSNitches within *TPT1* and *LCP1* mRNAs

We analyzed 37 allele-sorted data sets using the novel experimental protocol illustrated in Figure 2. We identified five riboSNitches using classSNitch in this data set (Woods and Laederach 2017). Our data are consistent with previous transcriptome-wide secondary structure analysis on a family trio that estimated that 15% of SNPs are likely riboSNitches (Wan et al. 2014). We find that 2/19 somatic mutations and 3/18 SNPs are riboSNitches with an overall ratio of 14% (Supplemental Table S1). A recent analysis of mutations within structured RNAs revealed significant differences in their susceptibility to mutation with an upper bound of over 60% of mutations disrupting the Lariat Capping Ribozyme structure (Woods and Laederach 2017). Nonetheless, on average 19% of mutations disrupted structure within these structured RNAs, suggesting that *TPT1* and *LCP1* structure are, on average, similarly robust to mutation as other RNAs. Furthermore, our data suggests that somatic mutations and inherited variation do not differ in their propensity for structural change. However, we do not find the UTRs or coding sequences to have a significant difference in riboSNitch frequency, implying that the likelihood of a point mutation changing a structure is dependent on the individual RNA's susceptibility to change (Woods and Laederach 2017).

We used the functional variant detection algorithm FATHMM-MKL to predict the consequences of the five riboSNitches we identified. Identifying which SNPs and mutations are biologically relevant is exceptionally difficult, and FATHMM-MKL is one of the few predictive algorithms to include noncoding mutations. FATHMM-MKL uses conservation, histone markers, and other features of the genome to assess whether a variant is likely to have a pathogenic effect. Scores are reported as *P*-values on a scale of 0–1, with higher numbers meaning more likely to be detrimental and lower numbers equating to neutral (Shihab et al. 2013, 2015). Although these predictive scores require experimental validation, FATHMM-MKL predictions perform well when compared with several databases of pathogenic mutations such as ClinVar and HGMD (Shihab et al. 2015). Four of the riboSNitches were predicted to be detrimental (Table 1). Within *LCP1,* the somatic riboSNitches COSM4526592 and COSM384608 had scores of 0.84 and 0.66, respectively, and, within *TPT1,* the inherited riboSNitches rs538915021 and rs11552475 had scores of 0.94 and 0.96 (Table 1). Although rs553866883 within *TPT1* had a neutral score (0.35), it is possible that its ability to change the structure of *TPT1* will make it more likely to be pathogenic in a manner not currently measured by FATHMM-MKL. FATHMM-MKL also predicted that many of nonchangers are harmful, as expected based on our growing knowledge of how synonymous and noncoding variations can influence pathogenesis even without altering RNA secondary structure (Supplemental Table S1; Hunt et al. 2014; Supek et al. 2014; Khurana et al. 2016).

**TABLE 1.**
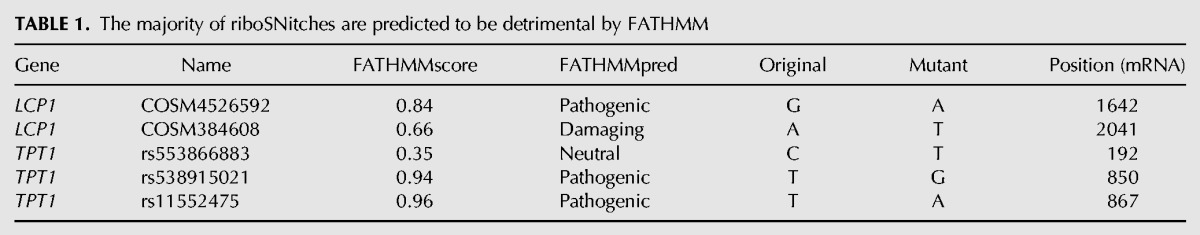
The majority of riboSNitches are predicted to be detrimental by FATHMM

We analyzed the structure change in all five riboSNitches by visualizing the ensemble of RNA structures. RNA is much more flexible than protein or DNA, usually existing as an ensemble of different structures instead of one stable conformation. We used the software ensemblerna to model and visualize these ensembles (Woods et al. 2017). Ensemblerna uses SHAPE data to guide a secondary structure folding algorithm. Then ensemblerna takes the set of structures, groups them, and counts the number of structures in each cluster before mapping the clusters onto a stable conformational space. To aid in understanding, we also show the medoid structure for the main clusters. Most riboSNitches cause a shift in the population of clusters and they sometimes cause formation of a new cluster. Due to sequencing restrictions we only obtained high depth information 100 nt around each mutation and may have missed larger structural effects from the five riboSNitches we detected. However, we expect the majority of structure changes to be local (Doshi et al. 2004; Kladwang et al. 2011a,b; Cordero et al. 2012; Woods and Laederach 2017).

### Somatic riboSNitches in the coding region of *LCP1* mRNA

**FIGURE 3.**
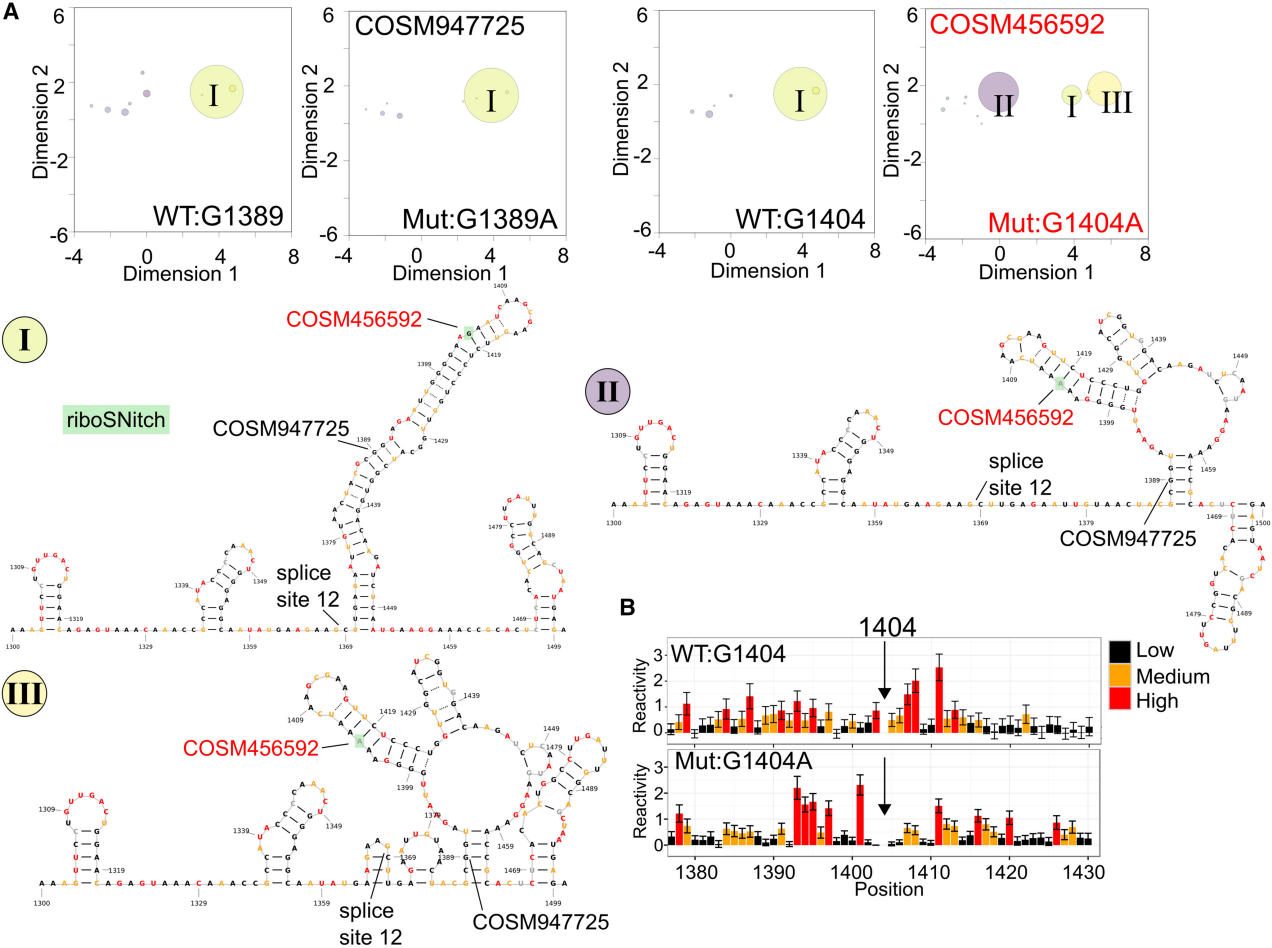
The riboSNitch COSM4526592 shifts the *LCP1* mRNA ensemble from one predominant structure group into three groups. (*A*) The SHAPE-guided structural ensembles for WT (G1389), Mutant (G1389A, nonchanger) and WT (G1404) have one main structure cluster (green bubble, *I*). The riboSNitch G1404A (COSM4526592), labeled in red, shifts the ensemble into two new structure clusters (purple, *II*; yellow, *III*). Each cluster is labeled in the ensemble plots (*A*) and illustrated with the medoid structures from the cluster (*I–III*). These medoid structures are example secondary structures for each cluster. The major allele structure is colored by WT SHAPE reactivity (*I*), whereas the nucleotides in the alternative conformations are colored by mutant reactivities (*II* and *III*). (*B*) Normalized SHAPE data around the riboSNitch show an increase in reactivity before the mutation and a decrease afterward in the mutant profile (bottom).

In the *LCP1* coding region (around position 1400), the mRNA folds as one group of related structures, shown with a representative secondary structure (Fig. 3A, large yellow bubble *I*). This group predominates within reference sequences and in another nonchanger mutation in the same region, COSM947725, which is highly likely to be neutral (FATHMM-MKL score: 0.06). However, the riboSNitch G1404A (COSM4526592), which is predicted to be pathogenic (FATHMM-MKL score: 0.84), causes a decrease in the population of the normal cluster and drives an increase in population of two additional clusters of structures (Fig. 3A, purple and light yellow bubbles *II* and *III*). The loss of a large dsRNA region is shown as the ensemble moves from the main WT cluster to alternative clusters (Fig. 3A). The normalized SHAPE data around the mutation site show a relatively local change with an increase in reactivity before the mutation site and a decrease in reactivity after the mutation site (Fig. 3B). We also analyzed the riboSNitch COSM384608 within the coding region of *LCP1*, which is predicted to be detrimental (FATHMM-MKL score: 0.66). Normally, this region exists as many different structure clusters (Supplemental Fig. S1A–C), but the riboSNitch COSM384608 causes a collapse of the ensemble into one main group (Supplemental Fig. S1D).

### Inherited rare variant riboSNitches in the 5′ and 3′ UTRs of *TPT1* mRNA

**FIGURE 4.**
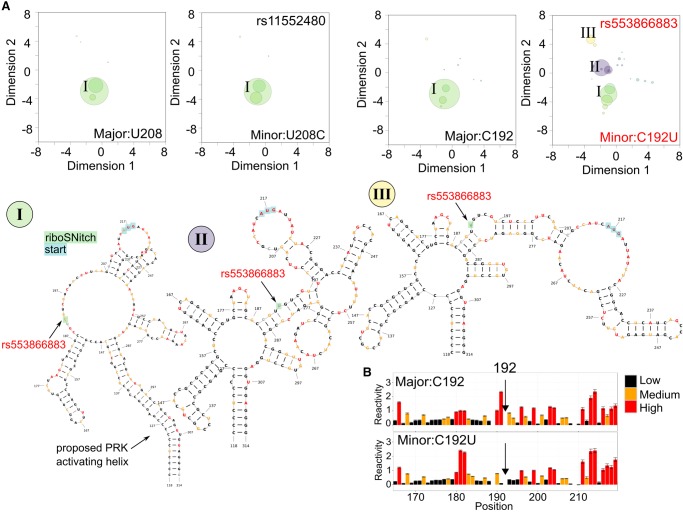
The riboSNitch rs553866883 shifts the *TPT1* mRNA ensemble from one predominant structure cluster into three. (*A*) The SHAPE-guided structural ensembles for major allele (U208), minor allele (U208C, rs11552489, nonchanger), major allele C192 all show one main structure cluster (green, *I*). The riboSNitch C192U (rs553866883), labeled in red, shifts the ensemble to form two new structure clusters that were previously not populated (purple, *II*; yellow, *III*). Each cluster is labeled in the ensemble plots and illustrated with the medoid structures from the group (*I–III*). The medoid secondary structure for the main cluster is colored by WT SHAPE reactivity while nucleotides in the alternative conformations (*II* and *III*) are colored by mutant reactivities. Points of references from the full-length structure are highlighted. (*B*) Comparison of normalized SHAPE data for the minor allele profile (*bottom*) compared to the major allele (*top*) around the riboSNitch.

Within the 5′ UTR of *TPT1*, there is one main group of similar structures (Fig. 4A, green bubble *I*). This region includes both the start codon and the location where PKR is predicted to bind TPT1 and cause translational repression. We observed this grouping of similar structures in mRNAs with the nonchanger SNV rs11552489 and with the major allele sequence for both the nonchanger and the riboSNitch rs553866883. However, the riboSNitch C192U (rs553866883) induces an increase in two additional clusters that are poorly populated in the major allele (Fig. 4A, bubbles *II* and *III*). Exemplary secondary structures from these clusters (Fig. 4A, *I*,*II*) indicate that the large dsRNA region preceding the variant has decreased in the alternative conformations and the variant itself moves from a relatively unstructured region into a more structured area.

The SHAPE data around the riboSNitch C192U indicate that this mutation causes a local change with a decrease in reactivity (increase in structure) before and after the SNP (Fig. 4B). The nonchanging SNV U208C (rs11552489) is predicted to be pathogenic (FATHMM-MKL score: 0.88), whereas the riboSNitch C192U (rs553866883) has a neutral FATHMM-MKL score (score: 0.35). In addition, two other nonchanger SNVs close by are also predicted to be pathogenic (rs776089085 and rs770667436, scores: 0.94 and 0.86) (Supplemental Table S1). We propose that the riboSNitch C192U (rs553866883) belongs to a group of SNVs that have functional importance within the 5′ UTR of *TPT1,* and they may not have been identified previously as pathogenic because of its cryptic effect on mRNA structure.

**FIGURE 5.**
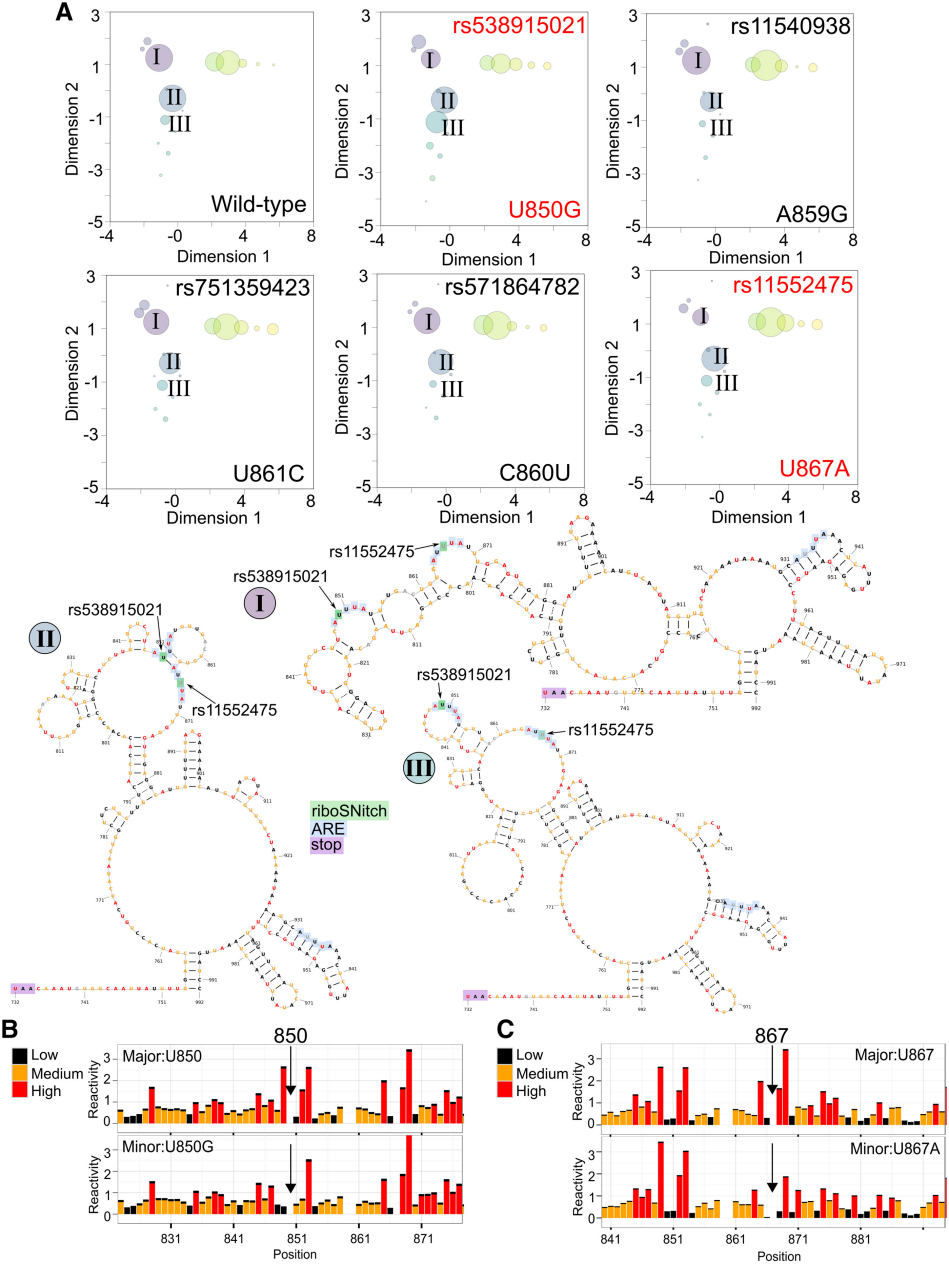
The riboSNitches rs538915021 and rs11552475 alter the proportions of the 3′ UTR *TPT1* mRNA structure clusters. (*A*) The SHAPE-guided structural ensemble for the reference sequence (control) is shown next to two riboSNitches (U850G and U867A). The riboSNitch U850G (rs538915021) causes an increase in population of the *III* cluster. The riboSNitch U867A (rs11552475) causes a decrease in the population of the *I* cluster. Three nonchangers are shown *below* (rs11540938, rs571864782, and rs751359423) that all occur within the same region. (*I–III*) Three representative medoid secondary structures are shown. Each cluster is labeled in the ensemble plots. The nucleotides in all structures are colored by WT SHAPE reactivity. (*B*,*C*) Comparison of normalized SHAPE data for the major and minor allele of the riboSNitches U850G (rs538915021) and U867A (rs11552475).

Two additional riboSNitches occur within the 3′ UTR of *TPT1,* which forms three main structure clusters that exist in relatively equal proportions (Fig. 5A, purple—*I*, blue—*II* and yellow bubbles) and one existing but less populated cluster (Fig. 5A, teal—*III*). The example medoid structures for the labeled structures illustrate the flexibility of this relatively open region (Fig. 5A). The riboSNitches U850G (rs538915021) and U867A (rs11552475) are both highly predicted to cause functional change to *TPT1* mRNA (FATHMM-MKL scores >0.94). RiboSNitch U850G (rs538915021) induces an increase in a secondary cluster that is not well populated in the reference (Fig. 5A bubble *III*), whereas riboSNitch U867A (rs11552475) induces a reduction in the main clusters (Fig. 5A bubble *I* and *III*). The SHAPE data for both riboSNitches indicate local change with a decrease in reactivity before or after the SNV, respectively (Fig. 5B,C). Three other SNVs within the same region do not alter the structural ensemble (Fig. 5A), but they are all predicted to be pathogenic (FATHMM-MKL scores >0.91) (Supplemental Table S1). The two riboSNitches occur within proposed AREs, and the other nonchangers are in close proximity. We measured the stability of *GFP-TPT1 3*′ *UTR* mRNAs with these variants and ARE-disrupting mutations, but we did not detect any differences (Supplemental Fig. S2). These AREs may be tissue specific and not functional in HEK-293 cells.

### The robust nature of highly structured *TPT1* mRNA

We performed probing experiments on *TPT1* to determine how the presence of proteins affects the *TPT1* structural ensemble. We reacted cellular mRNAs with the 1M7 SHAPE chemical probe that reacts with accessible 2′ hydroxyl groups and compared reactivity rates to a background (solvent-only) control (Wilkinson et al. 2006; Siegfried et al. 2014). To mimic the cellular environment, but maximize the reactivity of the probe, we treated cellular lysates with 1M7 and compared the results with mRNAs treated after extraction and in the absence of normal RNA-binding proteins. We also in vitro transcribed *TPT1* mRNA and compared the structural results of our protein-bound mRNA to artificially generated mRNA in the absence of any RNA-binding proteins. We used the SHAPE-MaP protocol in which SHAPE adducts are converted to mutations during library preparation, and the locations of adducts are identified as mutations through next-generation sequencing (Siegfried et al. 2014; Smola et al. 2015b). The SHAPE-MaP data sets used to create our secondary structure models for *TPT1* are available in the supplement (Supplemental Figs. S1–S3; Supplemental Files S1–S4, http://bit.ly/2sXWL3K; Sansone et al. 2012).

**FIGURE 6.**
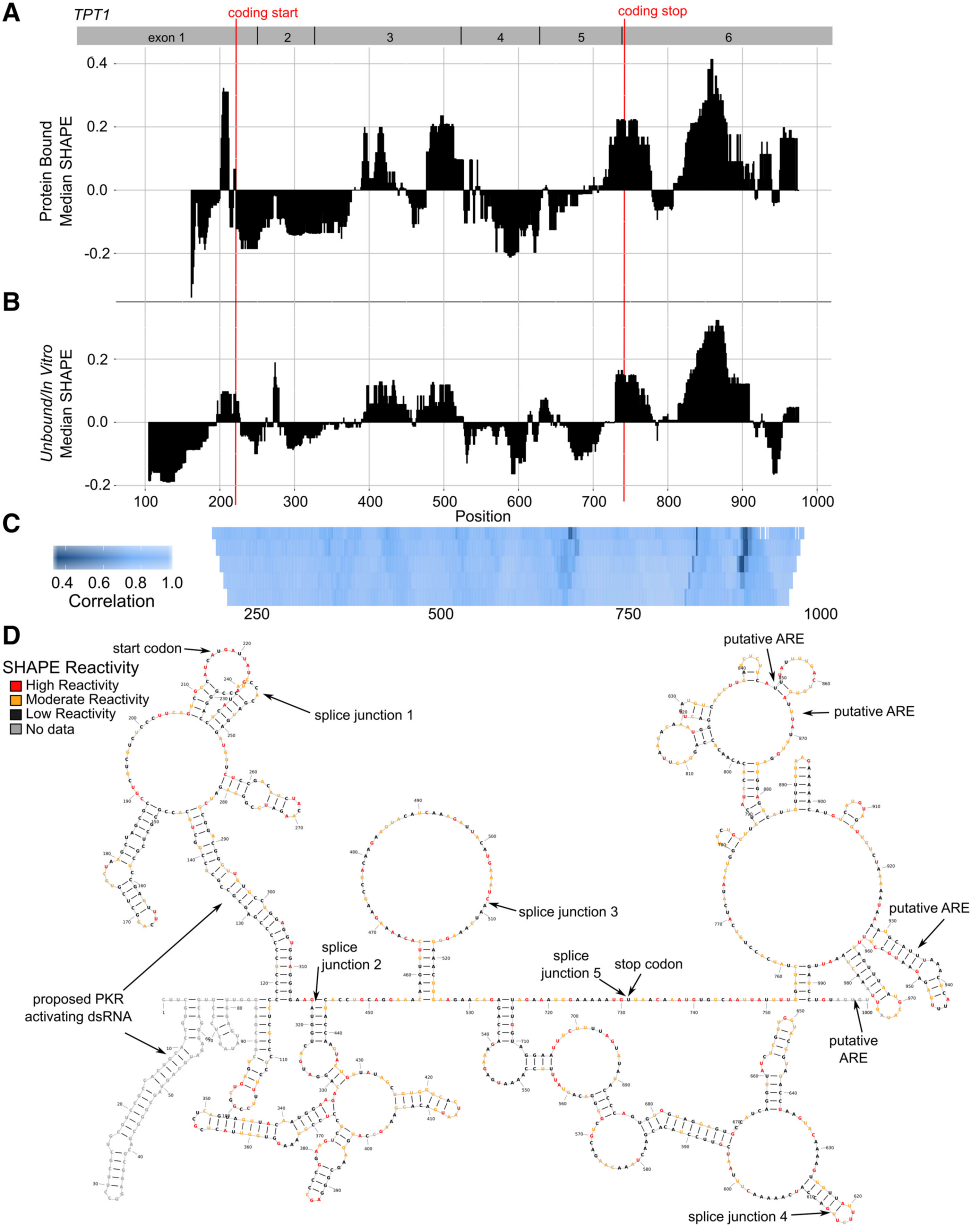
*TPT1* mRNA secondary structures are similar in the presence and absence of protein. (*A*) Median reactivity data from RNA in cellular lysate over a moving window of 40 nt indicating structured (low median SHAPE) and unstructured regions (high median SHAPE) within corresponds closely with the pattern of (*B*) median reactivity data from naked RNA. A 5′ to 3′ decrease in median SHAPE is observed, consistent with a highly structured 5′ UTR and less structured 3′ UTR. (*C*) Supportive of the overall similar median reactivities, pattern correlation of SHAPE reactivity is high (>0.85 overall). We performed correlations over multiple windows, from 10 to 50, *bottom* to *top*, with lighter blue indicating higher correlation. Regions of difference are noticeable as darker bands in the CDS and 3′ UTR. (*D*) *TPT1* secondary structure model informed with SHAPE reactivies from unbound RNA modification. Regions of interest are labeled, including the start codon, stop codon, proposed PKR activating helices in the 5′ UTR, and putative AREs in the 3′ UTR.

*TPT1* was originally characterized as a sequestered mRNA that is translationally induced during growth conditions (Gross et al. 1989). *TPT1* mRNA activates the double-strand RNA recognition protein PKR leading to repression of TPT1 protein translation, presumably through extensive double-stranded RNA structures within the 5′ UTR (Bommer et al. 2002; Nussbaum et al. 2002). *TPT1* is over-expressed in cancers and, although the mechanism is not fully elucidated, its protein product is believed to function as an antiapoptotic factor controlled by mTORC1 (Amson et al. 2013; Chen et al. 2013; Acunzo et al. 2014; Bommer et al. 2015; Thébault et al. 2016). We mapped the median SHAPE reactivity across *TPT1* to determine regions of high and low reactivity for the protein bound RNP (Fig. 6A) and naked RNA (Fig. 6B) conditions. This approach to visualizing the data enables us to identify regions of relatively high median SHAPE (less structure, more complex conformational ensemble) and relatively low median SHAPE (more structure, more likely a single conformation) (Smola et al. 2016). We treated the protein:mRNA complex with 1M7 (bound), extracted RNA from cells, removing all proteins (unbound), or we transcribed RNA with T7 RNA polymerase and then treated with 1M7 (in vitro) (Supplemental Figs. S3, S4). The unbound and in vitro samples had high agreement, as did all replicates (Supplemental Fig. S3D,O), allowing us to develop a robust structural model by merging and averaging the data to form a comprehensive in vitro SHAPE over nearly the entire transcript (Fig. 6D). We summarize further specifics of our correlation analysis in Supplemental Figures S3 and S5 and Supplemental Table S1. In general, the median reactivities for protein-bound, unbound and in vitro transcribed mRNA followed the same profile across *TPT1*, as corroborated by high levels of correlation between the samples, with an average correlation around 0.85 (Fig. 6C). For *TPT1* mRNA, we conclude that the overall fold of the mRNA is similar in all conditions examined, but specific smaller regions adopt different conformations based on conditions.

Using a model secondary structure informed with extensive in vitro SHAPE data, we found that *TPT1* is highly structured in the 5′ UTR, with more than 60% of the bases involved in pairing, and it is relatively open in the 3′ UTR, with less than 40% of the bases involved in base-pairing interactions. This observation correlates with the 5′ to 3′ increase in median SHAPE values plotted in Figure 6B. *TPT1* mRNA activates the double-stranded RNA (dsRNA) recognition protein PKR (Bommer et al. 2002; Nussbaum et al. 2002). PKR primarily recognizes viral dsRNA and inhibits viral translation, but PKR also interacts with a variety of cellular dsRNA-containing mRNAs, including the *TNF-*α and *IFN-*γ (Osman et al. 1999; Cohen-Chalamish et al. 2009; Hull and Bevilacqua 2016). Activation of PKR depends on a dsRNA region of at least 30 bases to allow binding of two or more PKR molecules (Manche et al. 1992; Zheng and Bevilacqua 2004; Lemaire et al. 2008). The highly structured 5′ UTR and first exon region of *TPT1* contain 95 base pairs (bp), primarily as two imperfect helices in close proximity, composed of 23 and 25 bp (Fig. 6D). This dsRNA region is likely to bind and activate PKR, resulting in translational inhibition of *TPT1* mRNA.

### Structured coding region of *LCP1* mRNA

**FIGURE 7.**
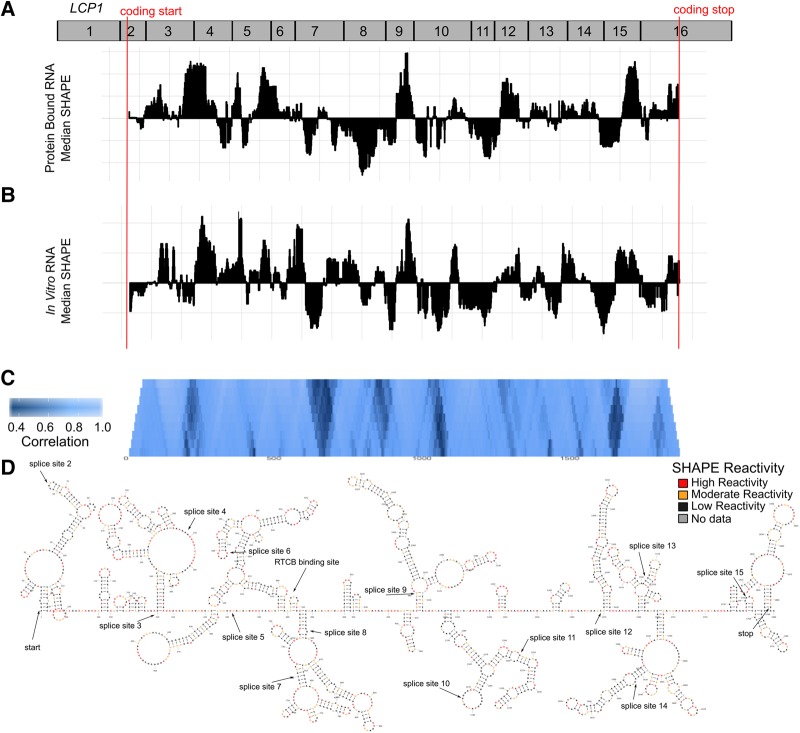
*LCP1* mRNA secondary structures are similar in the presence and absence of protein. (*A*) *LCP1* RNPs were probed and analyzed to generate median reactivity data. Low SHAPE regions indicate likely structured regions, while high SHAPE regions are likely unstructured. The median reactivity within the CDS of *LCP1* corresponds closely with the pattern of (*B*) naked RNA reactivity data. (*C*) The pattern of SHAPE reactivity is highly correlated (>0.82 overall). Correlations are performed over multiple windows, from 10 to 100, and lighter blue indicates higher correlation. Various regions of difference can be seen throughout the CDS as darker bands. (*D*) *LCP1* secondary structure model for the entire 1884 nt CDS using SHAPE data from unbound RNA to direct RNA structure prediction. Regions of interest are labeled, including splice junctions and potential RNA-binding protein sites.

*LCP1* is over-expressed in many different cancers and may be involved in cell mobility (Shinomiya 2012; Van Audenhove et al. 2016). Although little is known about the molecular mechanisms that regulate *LCP1* transcription and translation, a variant in the promoter region of *LCP1* causes a decreased risk for prostrate cancer (Chen et al. 2016) while other variants in *LCP1* are proposed as biomarkers for colorectal cancer recurrence and eQTLs (Garge et al. 2010; Ning et al. 2014). We performed probing experiments on *LCP1* mRNA to determine how the presence of proteins affects structural ensemble and how dependent *LCP1* structure is upon cellular conditions. We treated the *LCP1* mRNA in complex with its native RNA-binding proteins with the 1M7 SHAPE reagent and compared the results with 1M7 treatment of in vitro transcribed *LCP1* mRNA (Fig. 7A,B). We used the SHAPE-MaP protocol where 1M7 adducts are converted to mutations during library preparation, and identified with next-generation sequencing (Siegfried et al. 2014; Smola et al. 2015b). An overview of the different conditions used and raw data histograms are summarized in Supplemental Figure S5, and raw data are available in Supplemental Files S3 and S4 (also available on http://bit.ly/2sXWL3K) (Sansone et al. 2012).

*LCP1* has similar overall structures under cellular-like conditions with protein complexes and under in vitro transcribed conditions (Fig. 7A–C). The median reactivity profiles for *LCP1* mRNA in these environments were highly correlated with an average correlation coefficient of 0.82 (Fig. 7C). We derived a SHAPE-informed secondary structure model with the comprehensive in vitro SHAPE data set for *LCP1* mRNA (Supplemental Fig. S7D). *LCP1* is structured, but it does not have as many contiguous helices as *TPT1*. In PAR-Clip analyses, only the RNA-binding protein RCTB was found to interact with the *LCP1* mRNA coding sequence, although AGO2, LIN28B, IGF2BP1, and MOV10 can interact with the UTRs (Yang et al. 2015). Due to its extensive structure, we expect that post-transcriptional regulation is important for *LCP1* function.

### Environmentally dependent mRNA structures

Differences between structures from the same RNA as measured on the bench versus within the cell have been attributed to RNA-binding proteins, *N*-6-methyl adenosine modification, and active cellular unwinding, such as that associated with translation (Rouskin et al. 2014; Smola et al. 2015b; Spitale et al. 2015). Thus, we expect differences between *TPT1* and *LCP1* mRNAs in a cellular-like environment versus mRNAs transcribed in vitro with only T7 polymerase. This expectation is demonstrated best in our *TPT1* data sets with the lowest average correlation occurring between protein-bound *TPT1* and in vitro transcribed *TPT1* (Supplemental Table S2). We observed that the structure of *TPT1* transcribed in the cell, but removed from the cellular environment before probing, was in between RNA structures derived from experiments with more cellular protein-bound *TPT1* and in vitro transcribed *TPT1* (Supplemental Table S2). Because *LCP1* is not highly expressed, we do not have extensive coverage of this mRNA from transcriptome-wide experiments, however, within regions of adequate depth, *LCP1* transcribed within the cell did correlate better with protein-bound *LCP1* mRNA (Supplemental Fig. S5K, orange line), whereas the T7-transcribed in vitro *LCP1* data were much less correlated (Supplemental Fig. S5K, maroon and red lines). The similarities between the natively transcribed mRNAs, with or without protein binding, imply that transient protein-induced structural changes are not the only factors involved in differences between cellular and in vitro RNA structure, and additional properties such as cotranscriptional folding or splicing-influenced structures may also be important (Jin et al. 2011; McManus and Graveley 2011; Lai et al. 2013; Watters et al. 2016b).

To better understand the importance of environmentally dependent structures, we looked at whether these regions fell into particular categories. We found no connection between environmentally dependent structures and regions of high or low structure, RNA-binding protein regions, or splice sites (Supplemental Table S3; Supplemental Fig. S6). We analyzed the conservation of *LCP1* and *TPT1* mRNA sequences using scored alignments of 100 vertebrate sequences from the UCSC Table Browser (Karolchik et al. 2004; Siepel et al. 2005). PhyloP scores detect both conserved (positive values) and accelerated evolution (negative values) at the level of individual bases (Pollard et al. 2010). Both *LCP1* and *TPT1* are conserved, as the majority of PhyloP scores are above zero (Supplemental Fig. S7A,B), although *LCP1* has more nucleotides undergoing accelerated evolution compared with *TPT1* (negative values, Supplemental Fig. S6B). When we compared regions with environmentally dependent structures with conservation phyloP scores we noticed that environmentally sensitive regions (pink) tended to be more conserved than insensitive regions (blue) in both *TPT1* and *LCP1* (Supplemental Fig. S7). This trend is also present when analyzed in reverse, i.e., nucleotides with high PhyloP and high conservation are, on average, more environmentally similar (Supplemental Table S3). Although not statistically significant, this correlation suggests that the most highly conserved regions appear to undergo the most significant conformational rearrangements. How cellular conditions impact RNA secondary structure is an active area of research (Ding et al. 2014; Rouskin et al. 2014; Spitale et al. 2015; Smola et al. 2016; Watters et al. 2016a), and conservation may be key to understanding just how the environment affects RNA structures.

## DISCUSSION

Natural and somatic noncoding variations have the potential to yield significant insight into structure–function relationships in eukaryotic transcripts (Wan et al. 2014; Solem et al. 2015). In some cases, variants affecting UTR structure form a riboSNitch and cause human disease phenotypes (Halvorsen et al. 2010; Martin et al. 2012; Jafarifar et al. 2014; Rogler et al. 2014; Kutchko et al. 2015). However, it is likely that the vast majority of natural and somatic genetic variation is phenotypically benign. We still have a poor understanding of how natural noncoding genetic variation affects specific mRNA structures, and we are limited in our ability to predict these changes computationally (Ritz et al. 2012; Corley et al. 2015). Chemical probing experiments, especially high-accuracy approaches like SHAPE-MaP, detect structured regions in RNAs directly and enable us to identify the small subset of somatic and inherited variants that cause structural changes. Identification of these riboSNitches will further our understanding of the changes in RNA structure and lead to understanding which mutations and riboSNitches are pathogenic.

The median SHAPE values for *TPT1* and *LCP1* (Fig. 6,7) reveal that significant regions of mRNA have low median values with and without protein binding, consistent with a high degree of structure. These SHAPE data fit with our understanding of RNA folding thermodynamics that suggest that bases will pair if they are complementary (Mathews et al. 1999). Classic folding experiments with scrambled sequences demonstrate that even random RNAs will adopt stable secondary structures in complementary regions (Woodside et al. 2006). Thus, it is not surprising to find structured regions even within the coding sequence of mRNAs. Still, we do not understand how somatic or inherited variation will affect the fold of an mRNA in either highly structured or disordered regions. Our data suggest that the overall structural effects of somatic and inherited mutations do not depend on the local degree of structuredness (as measured by local median SHAPE, Supplemental Fig. S6) or functional context (UTR versus coding sequence, or even ARE). In fact, the majority of variants have no measurable effect on mRNA structure; consistently only around 15% of variants, either naturally occurring or man-made, resulted in even local structure change (Woods and Laederach 2017). Thus, the global folds of the *TPT1* and *LCP1* mRNAs appear relatively robust to variation. This insensitivity to variation contrasts with more highly structured RNAs such as elements of the ribosome and two ribozymes, for which recent analogous mutational analyses revealed significantly higher rates of structural disruption (over 60% for the Lariat Capping Ribozyme [Cheng et al. 2015; Woods and Laederach 2017]).

The *LCP1* and *TPT1* folds are also remarkably consistent between environmental conditions. Our data suggest that the fundamental structural features observed in vitro are preserved in the cell. The SHAPE-MaP approach improves the signal-to-noise by sequencing deeper and rigorously defines differences between protein-bound mRNA structures and in vitro mRNA folding. The extent of RNA structure change between cellular and in vitro conditions has been controversial and varies widely between studies and RNAs (Ding et al. 2014; Rouskin et al. 2014; Spitale et al. 2015; Watters et al. 2016a). We do identify multiple regions that are highly sensitive to environmental conditions (Figs. 6, 7; Supplemental Fig. S7) in both *TPT1* and *LCP1* mRNAs, and these regions tend to occur in regions with higher sequence conservation. The cellular environment causes differences between in vivo and in vitro SHAPE reactivity by rearranging the RNA structure, often through binding of RNA-binding proteins but also through many other mechanisms (Jin et al. 2011; McManus and Graveley 2011; Lai et al. 2013; Rouskin et al. 2014; Smola et al. 2015b; Spitale et al. 2015; Watters et al. 2016b). Our observation that these regions have higher sequence conservation suggests that interactions with the cellular environment may exert selective pressure on the RNA sequence. However, these differences do not occur with higher frequency in regions with low or high SHAPE reactivity (Supplemental Table S3), suggesting that interactions with the cellular environment are not limited to regions folding to single, well-defined conformations that have low median SHAPE (Smola et al. 2016).

We have shown that dissection of the molecular details of any mRNA requires high-resolution analysis using techniques such as allele-specific SHAPE-MaP that offer sufficient signal-to-noise (Fig. 2D) to infer specific structural ensemble models (Figs. 3–5). Furthermore, at present, SHAPE is the only structure probing technique for which there exists a validated, nucleotide resolution, thermodynamic correction for the nearest-neighbor rules (Deigan et al. 2009; Hajdin et al. 2013). We are beginning to unravel the intricacies of mRNA structural ensembles by performing allele-specific sorting and obtaining SHAPE ensemble models for a riboSNitch in single, clone-free experiments. The ensembles we visualize (Figs. 3–5) reveal the significant complexity of mRNA structures and the challenges faced when attempting to understand structure/function relationships in these regions. Furthermore, it is evident that specific variants and somatic mutations will alter the ensemble. The experimental and computational tools we propose and which we have used in this study provide a rigorous approach to dissect the complex interplay of ensemble thermodynamics, sequence selection, and RNA structure in driving noncoding function in the transcriptome.

## MATERIALS AND METHODS

### High-throughput multiplexed SHAPE treatment

Our modified high-throughput SHAPE-MaP protocol was performed on a Tecan Freedom Evo. We purchased mRNA clones of *TPT1* and *LCP1* (Origene—SC323772 and SC118739) and designed primers to introduce variants into select regions (Supplemental Table S6). Site-directed mutagenesis was performed with an NEB Q5 Site-Directed Mutagenesis Kit, but without customary transformation or cloning. Instead, we PCR-amplified the site-directed target after ligation using primers spanning the entire mRNA; the forward primer included a T7 promoter (NEB Q5 Hotstart). Ampure bead purification was performed to purify the DNA (Beckman Coulter—Ampure XP). Then we performed in vitro transcription with the T7 polymerase to synthesize RNA (NEB T7 Polymerase). To remove DNA, the sample was treated with TurboDNAse for 15 min at 37°C (ThermoFisher Scientific TurboDNAse Kit). Ampure bead purification was performed to purify the RNA (Beckman Coulter—RNAClean XP). To fold the RNA, samples were incubated at 37°C for 10 min in buffer containing 100 mM Na-HEPES, pH 8.0, 100 mM NaCl, and 10 mM MgCl_2_. The RNA was incubated for 5 min at 37°C with 10% dimethyl sulfoxide (DMSO) or with 10 mM 1-methyl-7-nitroisatoic anhydride (1M7) in DMSO. Ampure bead purification was performed to purify the modified RNA (Beckman Coulter—RNAclean XP).

### Selection of variants and their functional significance

We obtained somatic mutations from the COSMIC database (v72) (Forbes et al. 2015) and designed primers to recreate all synonymous variants within *TPT1* and *LCP1* (Supplemental Table S6). Inherited SNVs within *TPT1* were obtained from dbSNP (Sherry et al. 2001), and primers were designed in the same manner (Supplemental Table S6). We used the FATHMM-MKL webserver to extract predicted functional significance from all tested variants in *TPT1* and *LCP1* (Shihab et al. 2013, 2015). FATHMM-MKL estimates significance based on a variety of factors including conservation, histone modifications, transcription factor binding sites, and open chromatin. High FATHMM-MKL *P*-values equate to more likely deleterious substitutions, while lower *P*-values are more likely to be neutral.

### Protein-bound mRNA SHAPE treatment

Protein-bound mRNA SHAPE MaP experiments were performed with lymphoblastoid cell lines (1000 Genomes cell lines—NA07037, NA12003, NA19098, NA19099) (1000 Genomes Project Consortium 2015) or Tet-Off HEK-293 cells (Clontech, Tet-off cell line). For mutant analysis of the 3′ UTR of TPT1, HEK-293 cells were transfected with WT or mutant GFP-TPT1 3′ UTR constructs (GenScript, Clontech pTRE-TIGHT, NEB Q5 Site-Directed Mutagenesis Kit). HEK-293 cells were washed in PBS, trypsinized, and resuspended in complete media. Lymphoblastoid cell lines were pelleted by centrifugation. For all cell types, approximately 50 million cells were resuspended in 1 mL of folding buffer (same buffer as in vitro SHAPE protocol) and supplemented with 400 U murine RNAse inhibitor (NEB). Cells briefly sonicated at 10% power for three 10-sec intervals (Fisher Scientific Sonic Dismembrator Model 500). The lysates were incubated at 37°C for 5 min and then immediately modified. SHAPE treatment was performed for a period of 5 min with an addition of DMSO or three separate additions of 1M7 with a final concentration of 30 mM 1M7, 10% DMSO. RNA was extracted from the lysates, DNAse digested and depleted of ribosomal RNA (ThermoFisher TRIzol, 5PRIME PhaseLock Heavy, Invitrogen Purelink RNA columns, ThermoFisher Purelink DNase Set, and ThermoFisher Scientific RiboMinus Eukaryote System v2 from Life Technologies).

### Extracted mRNA SHAPE treatment

SHAPE-MaP experiments were performed with the same cell lines (above) on natively transcribed mRNAs. RNA was extracted by standard TRIzol purification and DNase digestion (ThermoFisher TRIzol, 5PRIME PhaseLock Heavy, Invitrogen Purelink RNA columns, ThermoFisher Purelink DNase Set) and incubated at 37°C for 10 min in folding buffer (above, in vitro SHAPE treatment). SHAPE treatment was performed for 5 min with an addition of DMSO or three separate additions of 1M7 with a final concentration of 30 mM 1M7, 10% DMSO. We performed buffer exchange (GE illustra MicroSpin G-50 Columns) before depletino of ribosomal RNA (RiboMinus Eukaryote System v2 from Life Technologies).

### Reverse transcription and library preparation

We performed SHAPE-Map reverse transcription with SuperScript II, random nonamers and error-prone conditions for all samples (Siegfried et al. 2014; Smola et al. 2015b) (ThermoFisher Scientific SuperScript II, NEB random nonamers). The samples were purified with Ampure XP beads or G-50 columns to isolate the cDNA (Beckman-Coulter, GE illustra MicroSpin G-50 Columns). For transcriptome-wide structure probing, we performed second strand synthesis (NEBNext Second Strand Synthesis Module) and either Nextera or Nextera XT library preparation samples (Nextera DNA Sample Preparation Kit, Nextera XT DNA Sample Preparation Kit and Index Kits from Illumina). For gene specific structure probing, we designed primers specific to the 3′ region, coding sequence, and 5′ region of the mRNA and PCR-amplified these regions after reverse transcription (NEB Q5 HotStart). We performed secondary PCR to add TruSeq barcodes. Sequencing for the T7 transcribed samples was performed on HiSeq2500 as paired end, 2 × 50 read multiplex run. Sequencing for the natively transcribed samples was performed on HiSeq2500 as paired end, 2 × 100 read multiplex runs. TruSeq libraries were sequenced as necessary for their designed length, primarily as paired end 2 × 300 read multiplex runs on a MiSeq instrument.

### SHAPE data analysis

For T7 transcribed samples and gene-specific TruSeq samples, we used bowtie2 (v2.2.9) to align SHAPE reads to either *LCP1* or *TPT1* mRNA (Langmead and Salzberg 2012). For transcriptome-wide experiments, we aligned reads to the entire genome (hg38). For riboSNitch analysis, we sorted the reads into WT or mutant based on the nucleotide at the mutation site, resulting in loss of SHAPE reactivity at the site of the mutation, but allowing us to separate the WT and mutant reads and determine overall reactivity for the entire region. The ShapeMapper pipeline to calculate mutation frequency has been previously described (Siegfried et al. 2014; Smola et al. 2015b). Briefly, we used the ShapeMapper algorithm to calculate the mutation frequency in the 1M7-treated sample, correcting for mutation frequency in the background (DMSO only) sample. We normalized the corrected reactivity by a multiplier based on the reactivity distribution of the full-length SERPINA1 transcript. To examine the broad, overall SHAPE reactivity, we averaged select data sets (defined in each section, available as Supplemental Files S1–S4 and on http://bit.ly/2sXWL3K) and calculated the median reactivity for 40–50 nt sliding windows (Pollom et al. 2013). We used this SHAPE reactivity to inform a minimum free energy structure using RNAstructure with a maximum pairing distance of 200/300 nt (Fig. 1; Supplemental Fig. S4). Incorporating SHAPE reactivities as a pseudo-free energy term in the nearest neighbor thermodynamic model of RNA folding improves the predictive capability of the model (Deigan et al. 2009; Hajdin et al. 2013; Sukosd et al. 2013).

### Identification of riboSNitches

To identify riboSNitches, we analyzed SHAPE traces for differences between WT controls and mutants manually and with classSNitch. Sequencing data were collected as 2 × 50 reads, thus, we restricted our analysis to 50–150 nt around the mutation. ClassSNitch uses a random forest algorithm based on expert classification to identify riboSNitches based on a comparison control and variant using normalized reactivities for each (Woods and Laederach 2017). To mimic a traditional between replicate analysis we compared the full-length data set for all nonchangers with allele-specific nonchangers (false positives) or allele-specific changers (true positives). To obtain the improvement offered by allele-specific sorting, we ratiometrically split each sample into 75% and 25% reads before calculating SHAPE reactivity. This splitting simulates the ratio of mutant to WT RNA modified and sequenced in the experiment, where there are more reads assigned to the mutant than to the WT. We compared these split reads to each other (no differences expected, false positives) and to split reads from manually identified riboSNitches (true positives). We used a ROC curve to quantify the improvement conferred by allele-specific sorting. To obtain the final set of riboSNitches we used allele-specific sorting with ClassSNitch. For riboSNitches within the 3′ UTR of *TPT1* we also performed a replicate-based experiment that agreed with the results of high quality allele-specific experiments. We identified five mutants as riboSNitches, all of which were verified by eye and the classSNitch algorithm (Table 1).

### Ensemble-based analysis of riboSNitches

We analyzed how the ensemble of RNA structures changed between the WT and mutant based on SHAPE-directed secondary structure predictions. Ensemblerna creates a conformational map of structures based on RNA sequence and visualizes them with multidimensional scaling that incorporates normalized SHAPE data, comparing the WT and mutant within the same space (Woods et al. 2017). Conformational maps were restricted to 50–200 nt of allele-specific data within the mutation, based on the sequencing data. The medoid structures for each cluster were used as representative secondary structure in all instances. All the classSNitch-identified riboSNitches showed differences in their secondary structure ensembles in mutant samples when compared with WT.

### RNA stability assay

We measured RNA stability with a doxycycline-inducible construct including GFP upstream of the 3′ UTR of TPT1 in a Tet-Off HEK-293 cell system (Genscript Gene Synthesis, Clontech pTRE-TIGHT) (Ysla et al. 2008). Tet-Off HEK293 cells were plated at a concentration of 150,000 cells/mL in Tet-free media supplemented with 100 μg/mL G418 followed by overnight incubation. Cells were transfected with WT of mutant 3′ UTR-GFP constructs using serum-free DMEM, 50 ng of construct, 350 ng of carrier DNA, and Transit (Mirus Transit). The cells were incubated for 24 h before the addition of 2 μg/mL of doxycycline. We sampled at 2, 4, and 6 h after addition of doxycycline and performed a standard TRIzol extraction to isolate RNA (Clontech Doxycycline, ThermoFisher TRIzol, 5PRIME PhaseLock Heavy, Invitrogen Purelink RNA columns). DNAse digestion and qRT-PCR were performed with the TaqMan Gene Expression Kit (Turbo DNAse, TaqMan Gene Expression kit) and TaqMan Gene Expression Probes from Applied BioSciences. We used probes that detected GAPDH and GFP (ThermoFisher Scientific, eGFP probe Mr04097229_mr and GAPDH probe Hs02758991_g1) on an ABI 7000 sequence detector system (Applied Biosystems). Data were analyzed by normalizing an average of triplicate samples with GAPDH, removing outliers, calculating expression, and comparing doxycycline-treated cells with untreated cells. Where ΔCT = GFP−GAPDH, the relative expression is (−ΔCT_dox_)^2^ ÷ (−ΔCT_untreated_)^2^ .

### Differences in RNA structure between environmental conditions

We downloaded both PhastCon and PhyloP-scored alignments of 100 vertebrates from the UCSC Table Browser (Karolchik et al. 2004; Siepel et al. 2005). We also downloaded PAR-Clip data for *TPT1* and *LCP1* from CLIP-db (Yang et al. 2015). Splice sites were defined as in the UCSC gtf files for each transcript (Karolchik et al. 2004). We defined highly similar ex vivo/in vitro regions as those with a correlation higher than 0.75 standard deviations from the mean and dissimilar regions as those with correlation lower than 0.75 standard deviations from the mean (40 nt window). We used bootstrapping to determine whether actual versus expected numbers of splice sites, RNA-binding protein sites, and conserved nucleotides occurred within similar or dissimilar regions of ex vivo/in vitro correlation. Other standard deviation groupings for highly similar or dissimilar nucleotides did not substantially change our correlations.

## SUPPLEMENTAL MATERIAL

Supplemental material is available for this article.

## Supplementary Material

Supplemental Material
