# Supplementary material for: Allele-specific SHAPE-MaP assessment of the effects of somatic variation and protein binding on mRNA structure
Source: RNA. 2018 Apr;24(4):513–28. doi: 10.1261/rna.064469.117 (PMC5855952; doi:10.1261/rna.064469.117)
Supplement: Supplemental Material [file supp_24_4_513__index.html]

Allele-specific SHAPE-MaP assessment of the effects of somatic variation and protein binding on mRNA structure — Supplemental Material 

# Allele-specific SHAPE-MaP assessment of the effects of somatic variation and protein binding on mRNA structure

## Supplemental Material

- Supplemental\_Figures\_and\_Tables.pdf
