# Supplementary material for: Allele-specific SHAPE-MaP assessment of the effects of somatic variation and protein binding on mRNA structure
Source: RNA. 2018 Apr;24(4):513–28. doi: 10.1261/rna.064469.117 (PMC5855952; doi:10.1261/rna.064469.117)
Supplement: Supplemental Material [file supp_064469.117_Supplemental_Figures_and_Tables.pdf]

Allele-specific SHAPE-MaP assessment of the effects of somatic variation on *TPT1* and *LCP1* mRNA structural ensembles

<u>**Supplemental Figures and Tables Contents:**</u>

Figure and Table Legends.

Supplemental Figures 1-7.

Supplemental Tables 1-4.

<u>**Supplemental Figures and Table Legends:**</u>

Supplemental Figure 1. The riboSNitch COSM384608 collapses the *LCP1* mRNA ensemble from many structure groups into one main cluster. (A) The wild-type 2041A has a diverse set of structures. An example secondary structure is shown for one of the main clusters (purple). (B) In contrast, the riboSNitch COSM384608 only has one predominate structure cluster. The mediod secondary structure is shown as a representation for this bubble (green). (C) Although no other mutants occur in this region, SHAPE data from the non-changers COSM151411 and COSM1941863 in the region of 2041 also indicate a diversity of structure groups.

Supplemental Figure 2. RiboSNitches and non-changers identified within the 3'UTR of *TPT1* do not alter RNA stability of *GFP-TPT1 3'UTR* mRNAs. (A) Construct schematic for the RNA stability system. TPT1 3'UTR sequence including four putative AREs, up to the second main polyA site, was inserted after the GFP coding sequence in a Tet-off promoter system (pTRE-TIGHT). All 3'UTR SNVs and ARE mutants (ATTTA/CTTTC) for all four putative AREs were created within the TPT1 3'UTR sequence. The constructs were transfected into Tet-Off HEK293 cells (ClonTech) and allowed to express for 24 hours before introduction of doxycycline. Cells were harvested in triplicate. (B) None of the tested constructs altered the stability of GFP-3'UTR TPT1. The wild-type sequence is shown in light blue with light blue ribbon indicating the error. We used qRT-PCR to quantify doxycycline treated and untreated cells with each construct at 2, 4, 6 and 8 hours. rs11540938 did have a consistently high RNA expression profile, but no change in stability. Only one ARE mutant was tested in this experiment, but previous experiments showed no effect with ARE1-3 mutants. Constructs marked with * are identified riboSNitches.

Supplemental Figure 3. Whole transcriptome and whole transcript (Nextera prepared) libraries used to obtain *TPT1* protein bound, unbound and *in vitro* secondary structure data sets. (A) Cells were sonicated to create lysate and then treated immediately with 1M7 before RNA extraction and library preparation. (B) SHAPE data across *TPT1* from protein bound CEU (NA07037 and NA12003) RNA. (C) Sequencing depth from the protein bound experiment within *TPT1.* (D) High correlation between protein bound RNP data (A-C) and the *in vitro (E-G)* and unbound data (H-J). (E) RNA was transcribed from linear double stranded RNA then treated with 1M7 before library preparation. (F) SHAPE data across *TPT1* from *in vitro* T7 transcribed RNA. (G) Sequencing depth from the *in vitro* experiment within *TPT1.* (H) RNA was extracted from cells with trizol (ribosomal RNA depleted) and then treated with 1M7 in the absence of proteins before library preparation. (I,K,M) SHAPE data across *TPT1* from naked

NA07037, NA19098 and NA19099 RNA respectively. (J,L,N) Sequencing depth from the NA07037, NA19098 and NA19099 naked RNA experiments within *TPT1.* (O) High correlation between naked cellular RNA replicates (NA07037, 19098 and 19099) across *TPT1.*

Supplemental Figure 4. Targeted SHAPE (TruSeq prepared) libraries used to obtain *TPT1* protein bound, unbound and *in vitro* secondary structure data sets. (A) HEK293 cells were transfected with GFP-TPT1 3'UTR expression plasmids then directly treated with 1M7 before RNA extraction and library preparation. (B) SHAPE data from the 3'UTR of *TPT1* obtained from protein bound RNA *GFP-TPT1* from transfected HEK293 cells. (C) Sequencing depth from the protein bound RNA experiment within *TPT1.* (D) High correlation between protein bound RNA data (A-C) and the naked RNA cellular data (E-G) obtained from *GFP-TPT1* RNA from transfected HEK293 cells. (E) HEK293 cells were transfected with GFP-TPT1 3'UTR expression plasmids and then RNA was extracted from cells with trizol and treated with 1M7 in the absence of proteins before library preparation. (F) SHAPE data across *TPT1* from cellular naked RNA. (G) Sequencing depth from the *ex vivo* experiment within *TPT1.* (G) Moderate correlation between the cellular naked RNA 3'UTR (E-G) data set and cellular naked RNA NA07037 whole transcriptome data (Supplemental Figure 3H-J). (i) RNA was extracted from cells with trizol (ribosomal RNA depleted) and then treated with 1M7 in the absence of proteins before library preparation. (J,L) SHAPE data within the 5'UTR of *TPT1* from cellular naked RNA from NA19098 and NA19099 cells respectively. (K,M) Sequencing depth from the NA19098 and NA19099 naked cellular RNA experiments. (n) High correlation between the naked cellular RNA 19098 and 19099 5'UTR data sets (purple) and moderate correlation between the naked cellular RNA 19098 5'UTR data set and the naked cellular RNA NA07037 transcriptome wide data set (Supplemental Figure 3H-J). (O,Q) SHAPE data within the CDS of *TPT1* from naked cellular RNA from NA19098 and HEK293 cells respectively. (P,R) Sequencing depth from the NA19098 and HEK2993 naked cellular RNA experiments. (S) High correlation between the naked cellular RNA CDS TruSeq replicates in different cell lines (O-R) as well as between naked cellular RNA CDS TruSeq NA19098 and naked cellular RNA NA19098 transcriptome wide data set (Supplemental Figure 3K-L).

Supplemental Figure 5. Data sets used to obtain *LCP1* protein bound and *in vitro* secondary structure. (A) Cells were lysed and then treated immediately with 1M7 before RNA extraction and library preparation. (B) TruSeq SHAPE data across the CDS of *LCP1* from protein bound HEK293 RNA. (C) Sequencing depth from protein bound HEK293 RNA. (D) *LCP1* RNA was transcribed from linear double stranded RNA then treated with 1M7 before library preparation. (E) SHAPE data across *LCP1* from *in vitro* T7 transcribed RNA. (F) Sequencing depth from the *in vitro* experiment. (G) RNA was extracted from cells with trizol (ribosomal RNA depleted) and then treated with 1M7 in the absence of proteins before library preparation. (H) SHAPE data across the CDS of *LCP1* from unbound CEU RNA (NA07037 and NA12003). (I) Sequencing depth from the CEU RNA experiment. (J) Correlation across *LCP1* between protein bound HEK293 RNA experiments and *in vitro* transcribed *LCP1.* (K) Correlation between 1100 and 1300 nt (within the CDS) of *LCP1* within a region of reasonable depth for the unbound transcriptome wide experiment (H-I) between unbound, protein bound (B-C) and *in vitro* (E-F) showing the difference between the *in vitro* sample when compared to protein bound or unbound RNA (maroon and red versus orange).

Supplemental Figure 6. Local structuredness does not predict whether a variant will change RNA structure. (a) Each point indicates the average SHAPE reactivity (+/- 5 nts) around a variant classified as a riboSNitch. The blue points are riboSNitches within *LCP1* and the orange points are riboSNitches within *TPT1*. Lines indicate the average SHAPE reactivity for both transcripts. (b) The average SHAPE reactivity (+/- 5 nucleotides) around nonchanger variants ranges from very structured (low values) to unstructured. The blue points are *LCP1* nonchangers and orange points are *TPT1* nonchangers. Lines indicate the average SHAPE reactivity for both transcripts. (c) Bootstrapping was used to look at 1000 instances of 17 random positions in *LCP1* for how many in each set were below the mean SHAPE value. The number of nonchangers in structured regions in *LCP1* is within the expected value (9 out of 17 nonchangers, red star). (d) Bootstrapping was used to look at 1000 instances of 15 random positions in *TPT1* for how many in each set were below the mean SHAPE value. The number of nonchangers in structured regions in *LCP1* is within the expected value (6 out of 15 nonchangers, red star).

Supplemental Figure 7. Conserved nucleotides tend to have different SHAPE reactivity between protein bound and unbound conditions. (A) The PhyloP conservation scores are indicated as dots for each nucleotide in *LCP1*; the majority are above zero indicating conservation (rapid evolution equates to negative values). Each dot is colored based on the correlation between the protein bound and unbound samples. The dark blue dots indicate that the SHAPE data around that nucleotide are insensitive to the presence of protein, whereas the pink dots indicate the SHAPE data are sensitive to the environment. Points falling in between are colored light blue, and nucleotides without correlation data are colored grey. (B) Nucleotides from *LCP1* that are sensitive to the presence of proteins (pink) shift toward more conserved phyloP scores compared to nucleotides that are insensitive to protein (blue). (C) Nearly all the nucleotides in *TPT1* are conserved (PhyloP scores above zero). Color scheme is as previously described in A. (D) Nucleotides in *TPT1* that are sensitive to the presence of proteins (pink) shift toward being more conserved when compared to nucleotides that are insensitive to the presence protein (blue).

Supplemental Table 1. ClassSnitch and manual analysis identified 5/37 riboSNitches (red, feature scores for the algorithm are included). The majority of riboSNitches as well as many non-changers are predicted to be detrimental by FATHMM.

Supplemental Table 2. Correlation between datasets used for TPT1 secondary structure model and correlation between conditions, cell lines and replicates.

Supplemental Table 3. More conserved (phyloP) nucleotides tend to be less similar in vivo to in vitro. There are no consistent differences between in vivo and in vitro SHAPE due to splice sites, RNA binding protein sites (PARclip) or level of structuredness.

Supplemental Table 4. Primers used for site-directed mutagenesis and PCR amplification of specified regions for in vitro RNA production or library generation.

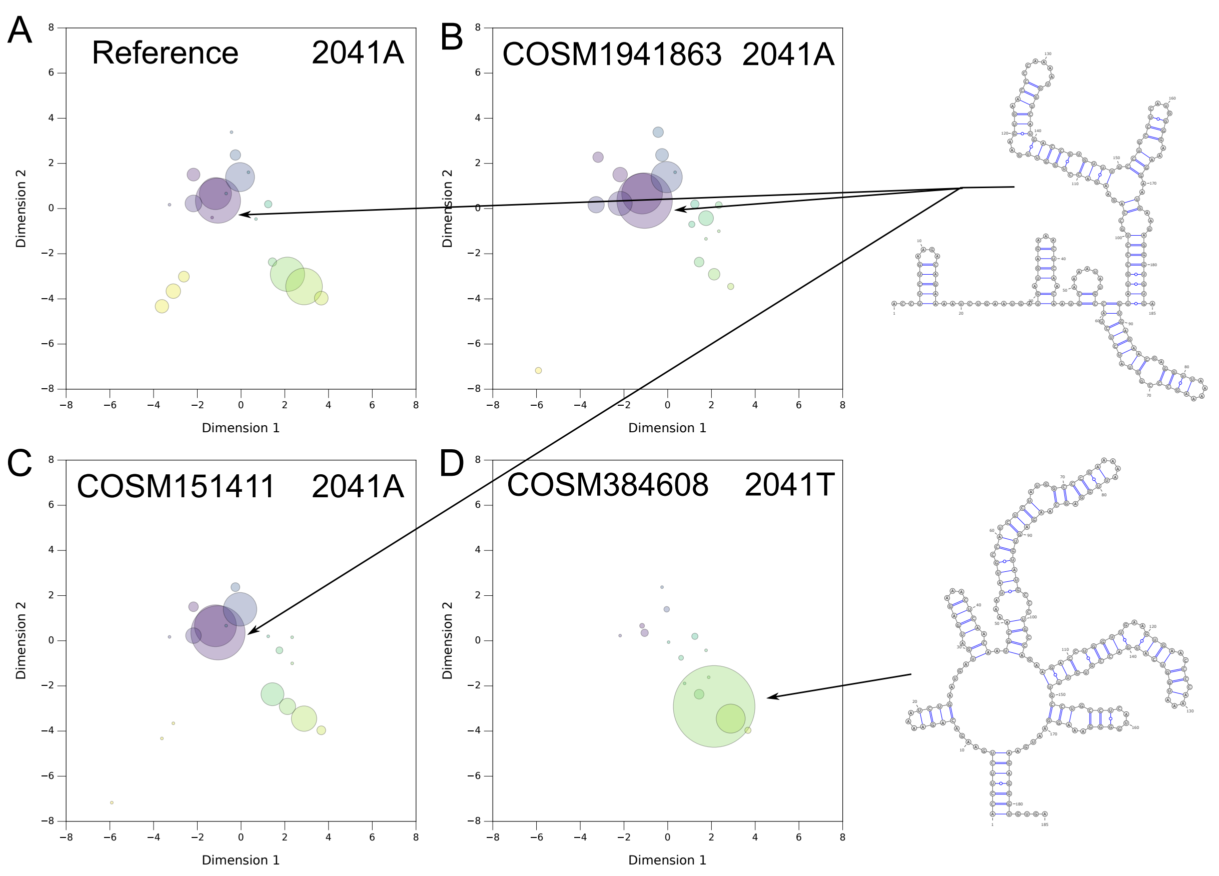

Supplemental Figure 1.

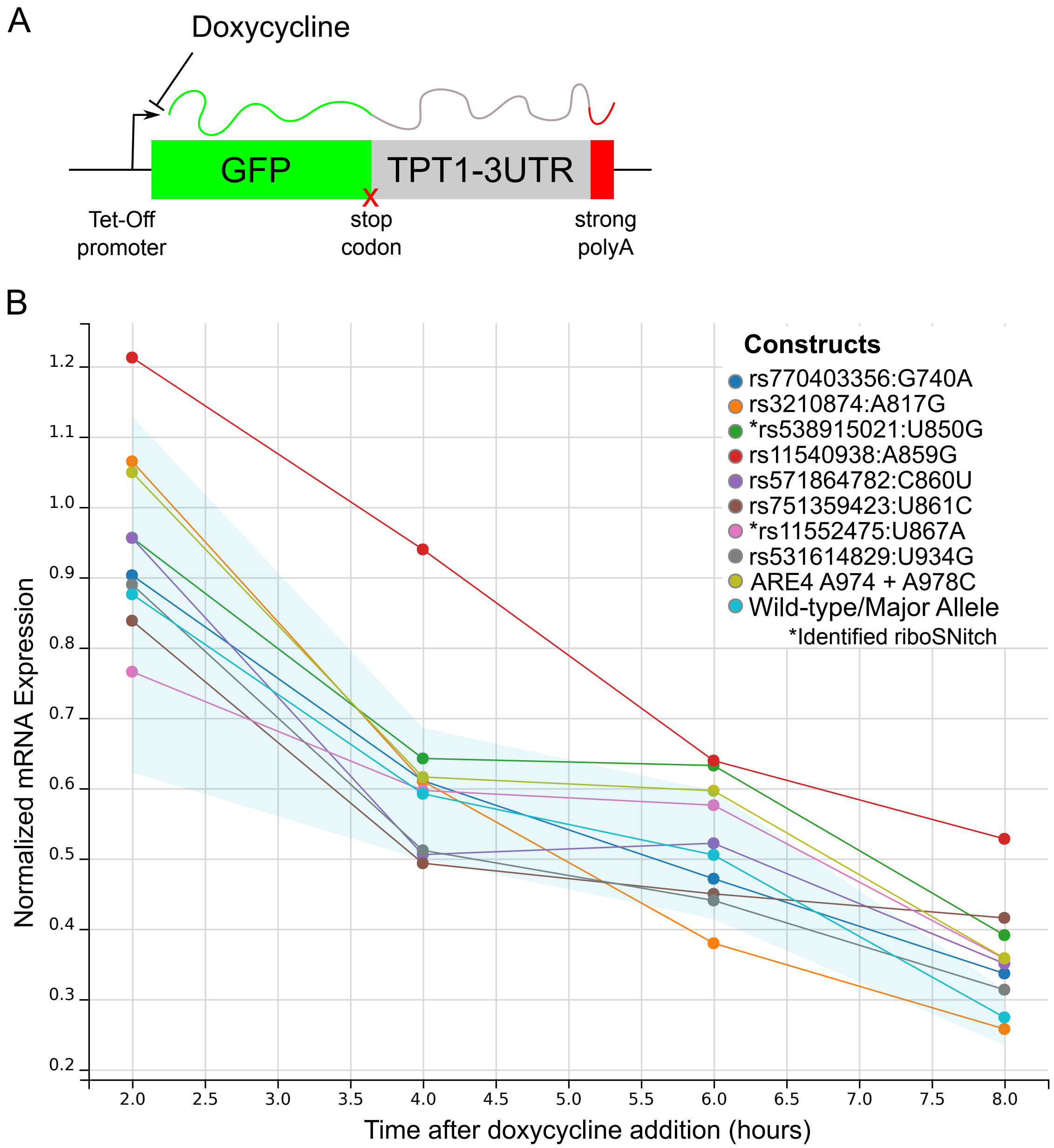

Supplemental Figure 2.

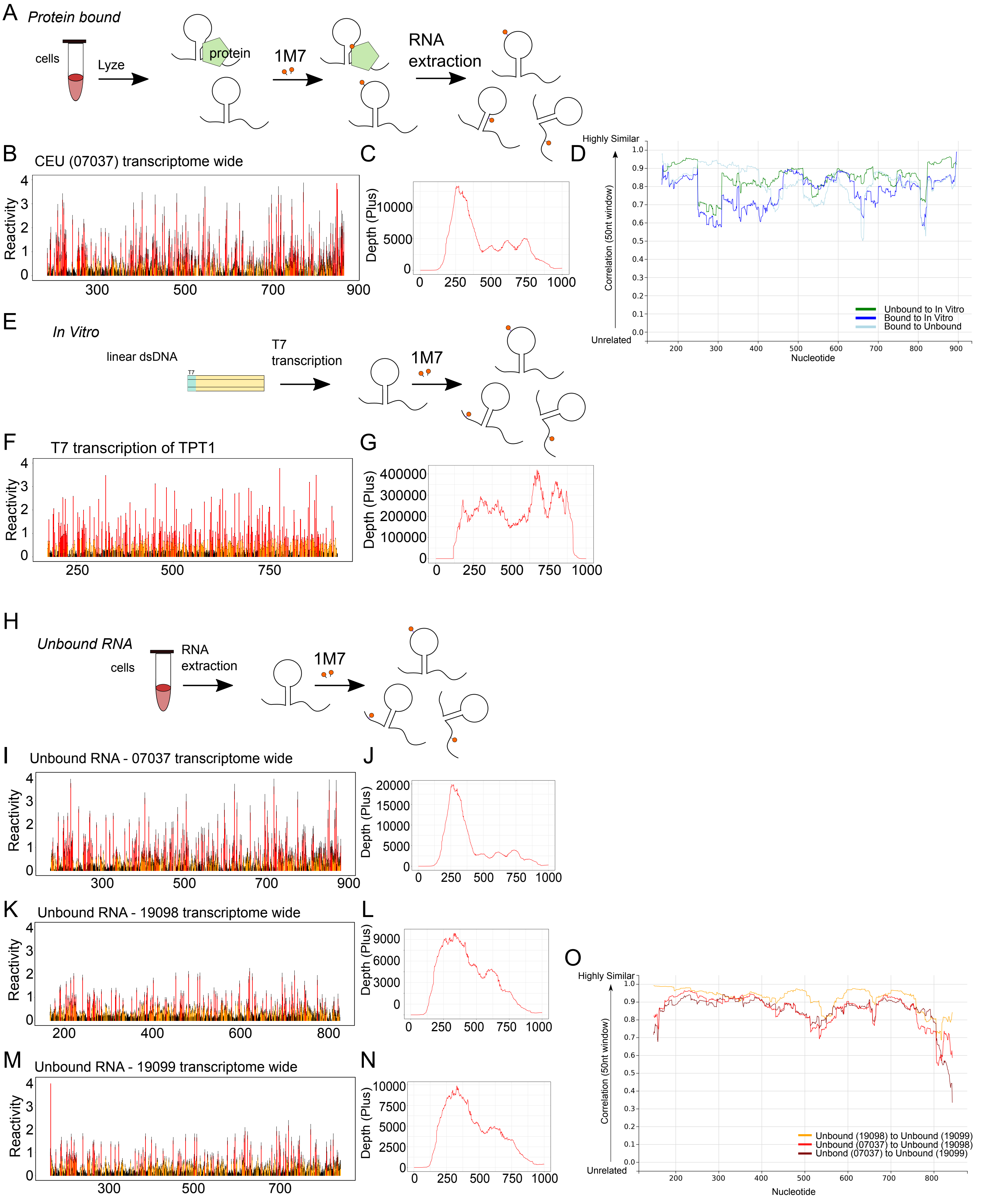

Supplemental Figure 3.

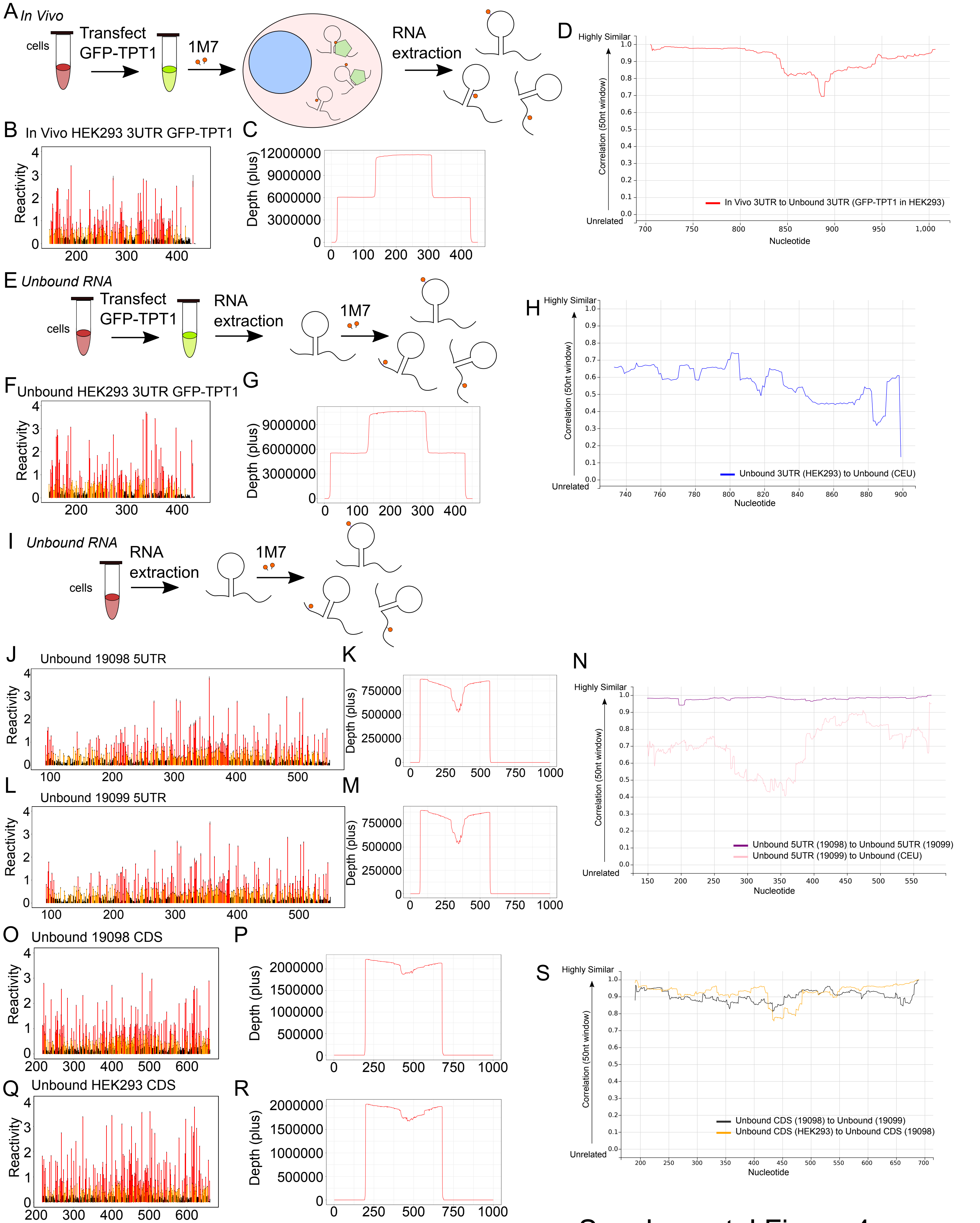

Supplemental Figure 4.

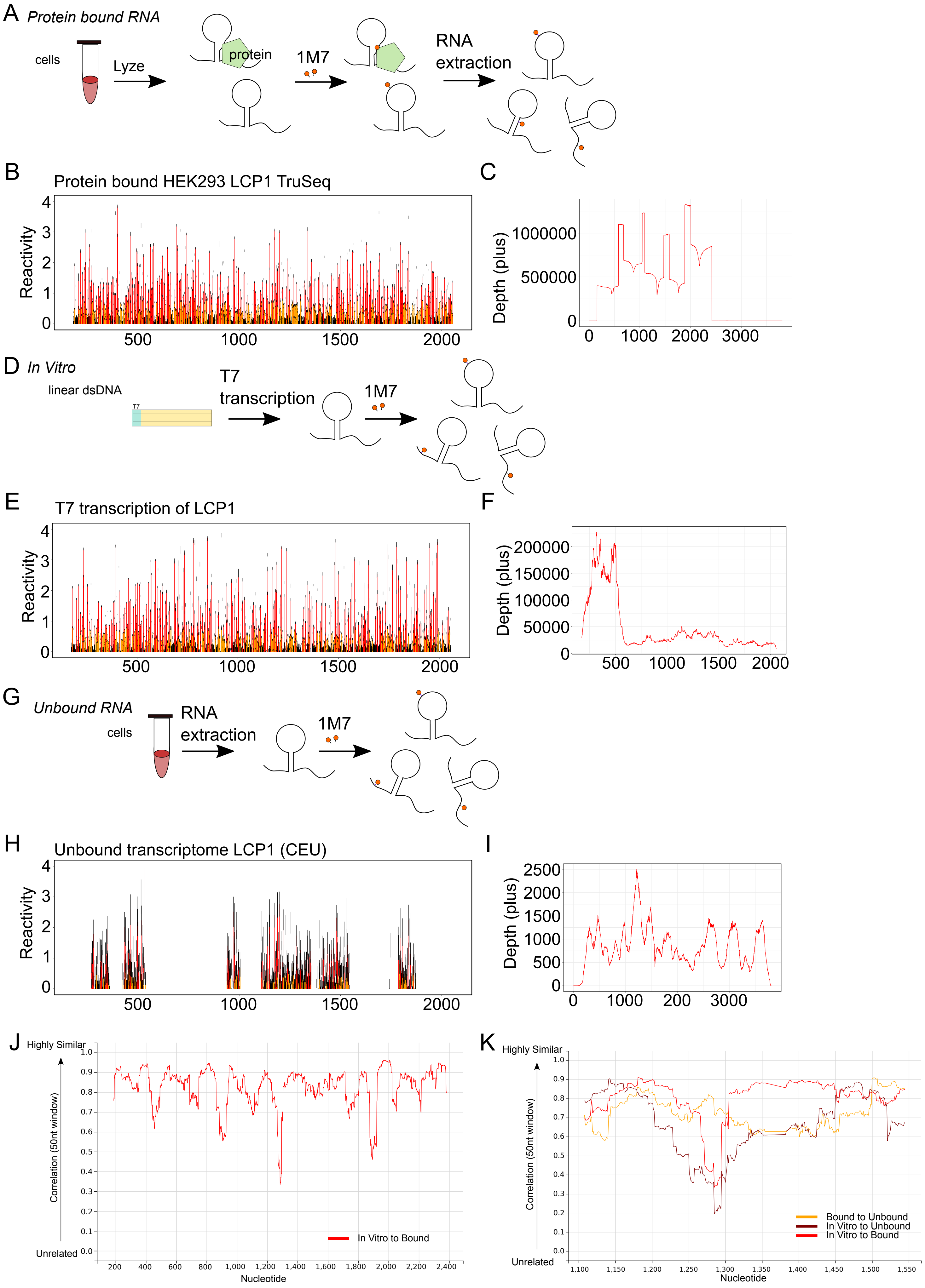

Supplemental Figure 5.

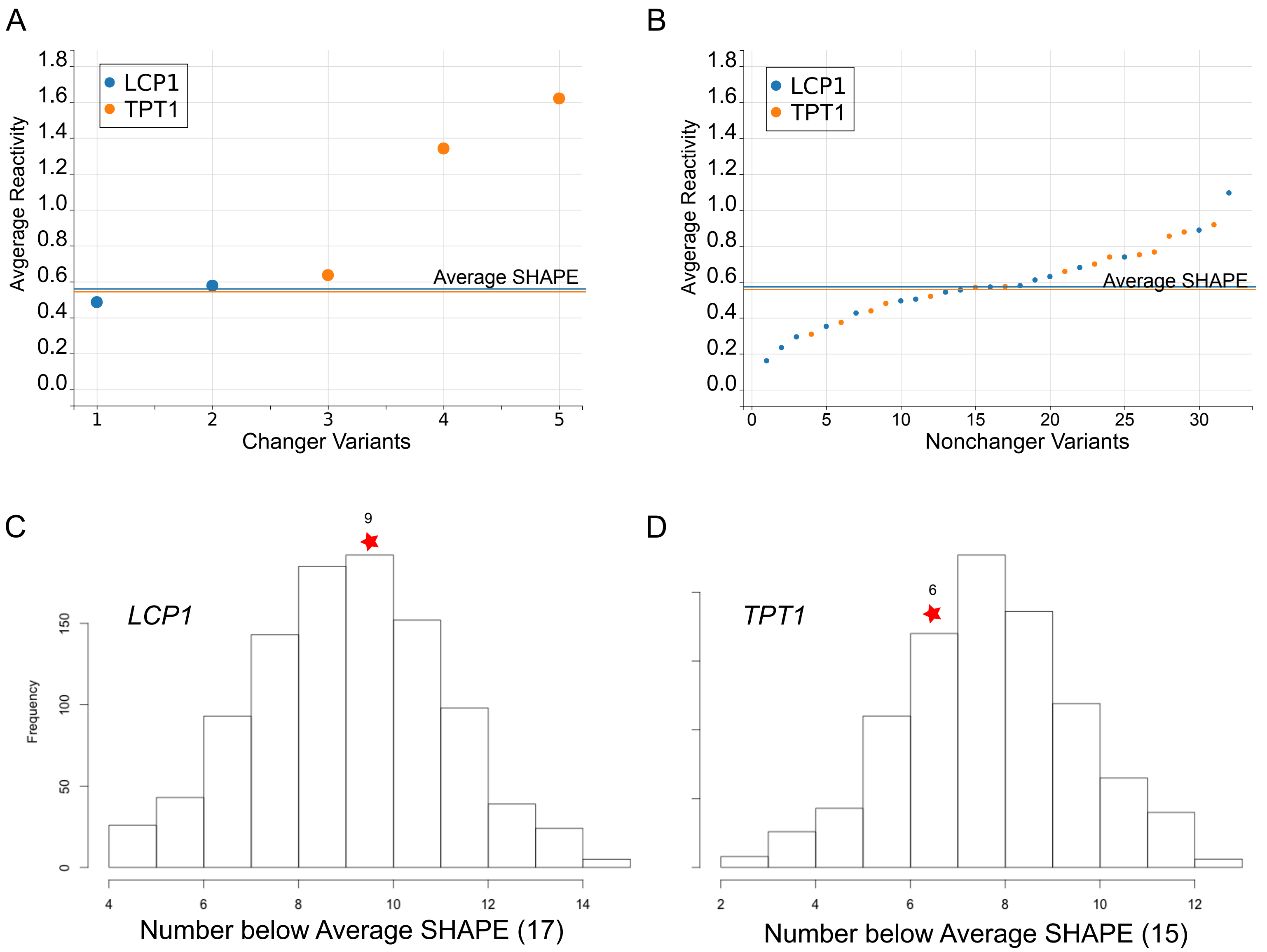

Supplemental Figure 6.

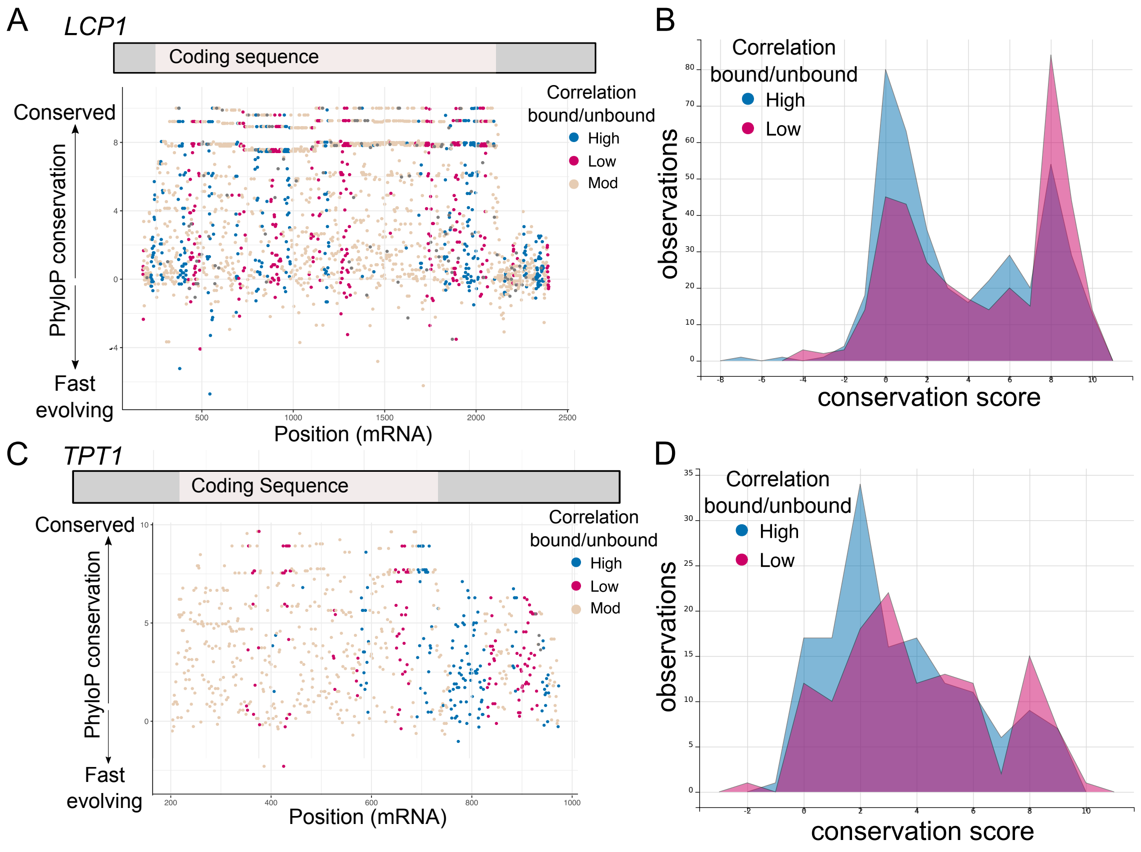

Supplemental Figure 7.

Supplemental Table 1.

Gene	RiboSNitch	Name	FATHMMscore	FATHMMpred	Original	Mutant	Position (mRNA)	Method	pattern change	DTW	Contiguous	Pearson CC	Variance	eSDC	Range	L2 norm
LCP1	No	COSM237651	0.90	Pathogenic	C	T	430	ClassSnitch	0.8302352	0.07278922	29	0.96069315	0.2414318	0.0781801	144	2.460734
LCP1	No	COSM1941863	0.91	Pathogenic	G	A	442	ClassSnitch	0.7881272	0.09650461	38	0.96006034	0.3153915	0.078128602	147	2.947705
LCP1	No	COSM80099/rs754437941	0.70	Pathogenic	C	A	577	ClassSnitch	0.5571257	0.12745085	68	0.93915866	0.5936913	0.076427648	148	3.434253
LCP1	No	COSM1514111	NA	NA	A	G	595	ClassSnitch	0.6557307	0.11627721	58	0.90022288	0.4878747	0.073259099	143	3.574423
LCP1	No	COSM332631	0.11	Neutral	G	A	661	ClassSnitch	0.6434828	0.12542605	54	0.89397107	0.5630425	0.072750334	149	3.238829
LCP1	No	COSM3688735	0.91	Pathogenic	C	T	718	ClassSnitch	0.658169	0.09642114	52	0.92124237	0.5704251	0.074969641	143	3.156108
LCP1	No	COSM3469102	0.06	Neutral	T	G	1003	Manual	N/A	N/A	N/A	N/A	N/A	N/A	N/A	N/A
LCP1	No	COSM4519740	0.85	Pathogenic	G	A	1264	Manual	N/A	N/A	N/A	N/A	N/A	N/A	N/A	N/A
LCP1	No	COSM4641819/rs770350377	0.25	Neutral	G	A	1573	ClassSnitch	0.6223315	0.10486093	53	0.91989015	0.5635794	0.074859599	143	2.919894
LCP1	No	COSM947725/rs748447395	0.06	Neutral	G	A	1627	ClassSnitch	0.6708824	0.0935386	50	0.93602598	0.5098881	0.076172714	146	2.558581
LCP1	Yes	COSM4526592	0.84	Pathogenic	G	A	1642	ClassSnitch	0.5114336	0.14784864	76	0.75060617	0.6473826	0.061083464	150	3.725266
LCP1	No	COSM81374/rs141184605	0.63	Damaging	G	A	1651	ClassSnitch	0.5205008	0.13897008	73	0.90140189	0.6364653	0.073355046	150	2.307838
LCP1	No	COSM4773990/rs766808077	0.71	Pathogenic	C	T	1730	ClassSnitch	0.461443	0.09489896	66	0.89817706	0.8635794	0.073092612	150	3.20823
LCP1	No	rs4941543	0.00	Neutral	A	G	1835	ClassSnitch	0.7221209	0.08402841	42	0.95139792	0.3959284	0.077423665	142	2.451564
LCP1	No	COSM3469097	0.98	Pathogenic	G	A	1864	ClassSnitch	0.7204401	0.10323107	43	0.92699992	0.3890828	0.075438184	149	2.891597
LCP1	No	rs11558762	0.07	Neutral	G	T	1870	ClassSnitch	0.7554498	0.10007537	42	0.91419195	0.3418792	0.074395886	137	3.031351
LCP1	No	COSM95983	0.77	Pathogenic	C	A	1927	ClassSnitch	0.4707596	0.14693989	80	0.85823763	0.6666667	0.069842388	148	3.089354
LCP1	Yes	COSM384608	0.66	Damaging	A	T	2041	ClassSnitch	0.4009089	0.08736523	27	0.94623428	0.8397306	0.126445874	54	1.507801
LCP1	No	COSM1743690/rs756230774	0.64	Damaging	C	G	2095	Manual	N/A	N/A	N/A	N/A	N/A	N/A	N/A	N/A
TPT1	No	rs780461001	0.30	Neutral	T	G	165	ClassSnitch	0.645	0.068	45	0.948	0.391	0.080	119	2.347
TPT1	No	rs751424907	0.92	Pathogenic	C	T	171	ClassSnitch	0.636	0.093	45	0.757	0.449	0.063	127	4.259
TPT1	No	rs763788350	0.77	Pathogenic	G	C	174	Manual	N/A	N/A	N/A	N/A	N/A	N/A	N/A	N/A
TPT1	No	rs752572384	0.29	Neutral	T	C	183	ClassSnitch	0.620	0.108	54	0.712	0.497	0.058	137	5.115
TPT1	No	rs776089085	0.94	Pathogenic	G	A	187	ClassSnitch	0.637	0.108	56	0.836	0.463	0.068	135	3.793
TPT1	No	rs770667436	0.86	Pathogenic	T	A	191	ClassSnitch	0.634	0.106	63	0.775	0.496	0.063	141	4.739
TPT1	Yes	rs553866883	0.35	Neutral	C	T	192	ClassSnitch	0.517	0.116	72	0.587	0.638	0.048	141	6.065
TPT1	No	rs11552489	0.88	Pathogenic	T	C	208	ClassSnitch	0.688	0.121	53	0.775	0.463	0.063	149	4.956
TPT1	No	COSM3369079	0.09	Neutral	C	T	425	ClassSnitch	0.802	0.089	37	0.901	0.327	0.073	130	3.041
TPT1	No	COSM4735650/rs755836071	0.68	Damaging	A	G	575	ClassSnitch	0.787	0.079	38	0.952	0.315	0.077	150	2.204
TPT1	No	rs770403356	0.35	Neutral	G	A	740	ClassSnitch	0.753	0.089	43	0.921	0.423	0.075	130	3.128
TPT1	No	rs3210874	0.91	Pathogenic	A	G	817	ClassSnitch	0.764	0.107	39	0.909	0.373	0.074	147	3.683
TPT1	Yes	rs538915021	0.94	Pathogenic	T	G	850	ClassSnitch	0.588	0.149	48	0.623	0.643	0.055	117	6.467
TPT1	No	rs11540938	0.94	Pathogenic	A	G	859	ClassSnitch	0.650	0.095	43	0.948	0.539	0.088	113	2.368
TPT1	No	rs571864782	0.94	Pathogenic	C	T	860	ClassSnitch	0.937	0.069	15	0.963	0.095	0.068	140	2.458
TPT1	No	rs751359423	0.91	Pathogenic	T	C	861	ClassSnitch	0.917	0.072	21	0.949	0.125	0.067	136	2.635
TPT1	Yes	rs11552475	0.96	Pathogenic	T	A	867	ClassSnitch	0.930	0.077	19	0.934	0.105	0.066	190	3.139
TPT1	No	rs531614829	0.95	Pathogenic	T	G	934	ClassSnitch	0.804	0.152	37	0.788	0.276	0.058	172	5.500

Supplemental Table 2.

Comparison	Importance	AvgCorr	StDevCorr
In Vivo (CEU) to In Vitro (T7 transcribed)	Condition comparison	0.77	0.09
Ex Vivo (CEU) to In Vitro (T7 transcribed)	Condition comparison	0.85	0.08
In Vivo (CEU) to Ex Vivo (CEU)	Condition comparison	0.82	0.08
Ex Vivo (CEU) to Ex Vivo (YRI)	Different cell lines	0.85	0.11
Ex Vivo (CEU) to Ex Vivo (YRI98)	Different cell lines	0.82	0.21
Ex Vivo (YRI98) to Ex Vivo (YRI99)	Different cell lines	0.92	0.09
5'UTR Ex Vivo (YRI98)* to 5'UTR Ex Vivo (YRI99)	Different cell lines	0.98	0.01
5'UTR Ex Vivo (YRI99)* to Ex Vivo (CEU)	Target PCR to whole transcriptome	0.70	0.15
CDS Ex Vivo (YRI98)* to Ex Vivo (YRI98)	Target PCR to whole transcriptome	0.90	0.03
CDS Ex Vivo (HEK293)* to CDS Ex Vivo (YRI98)*	Different cell lines	0.93	0.05
3'UTR In Vivo (HEK293)* to 3'UTR Ex Vivo (HEK	Condition comparison	0.92	0.07
Ex Vivo (CEU) to 3'UTR Ex Vivo (HEK293)*	Different cell lines, Target PCR to whole transcriptome	0.54	0.20

*Tru-Seq PCR amplified library (as opposed to Nextera prepared)

Supplemental Table 3.

Gene	Tested Variable	Mean Correlation to In Vivo/Vitro	SD
LCP1	Less Conserved	0.835	0.113
LCP1	More Conserved	0.811	0.127
TPT1	Less Conserved	0.874	0.073
TPT1	More Conserved	0.870	0.055
LCP1	Splice Sites	0.842	-
LCP1	Simulated Splice Sites	0.825	0.030
TPT1	Splice Sites	0.879	-
TPT1	Simulated Splice Sites	0.878	0.025
LCP1	PARClip Sites	0.877	-
LCP1	Simulated PARclip Sites	0.773	0.824
TPT1	PARClip Sites	0.883	-
TPT1	Simulated PARclip Sites	0.880	0.005
LCP1	High SHAPE	0.836	0.112
LCP1	Moderate SHAPE	0.817	0.123
LCP1	Low SHAPE	0.825	0.119
TPT1	High SHAPE	0.875	0.058
TPT1	Moderate SHAPE	0.867	0.070
TPT1	Low SHAPE	0.879	0.065

Supplemental Table 4.

Gene	Targeted Mutation	Sequence
LCP1	192C>T	GTG ATC TGG ATC AAG ATG GAA G
LCP1	204G>A	AAG ATG GAA GAA TCA GCT TTG ATG
LCP1	339C>A	CTA GCG TTG GAA CCC AAC ACT
LCP1	357A>G	ACT CCT ATT CGG AGG AAG AAA AGT ATG
LCP1	423G>A	CTG ATT GTC GAC ATG TCA TCC
LCP1	480C>T	TTG GAG ATG GTA TTG TCC TTT G
LCP1	765T>G	TGA GAG AAG GGG AGA GCC TGG
LCP1	1026G>A	AGC AGG CGG AAA GGC TGG GCT
LCP1	1224T>C	ACT CCC TGG GCG TTA ACC CTC
LCP1	1335G>A	TAA ACA AAC CAC CAT ACC CCA AAC
LCP1	1389G>A	GTA ACT ACG CAG TAG AAT TGG G
LCP1	1404G>A	AAT TGG GGA AAA ATC AAG CGA AG
LCP1	1413G>A	AGA ATC AAG CAA AGT TCT CCC TGG
LCP1	1492C>T	GAT TTG GCA GTT AAT GAG AAG GTA TAC
LCP1	1518C>T	CAC TGA ATA TTC TCG AAG AAA TTG
LCP1	1539C>T	TTG GTG GTG GTC AGA AGG TCA
LCP1	1575G>A	TCA ACT GGG TAA ATG AAA CAT TG
LCP1	1626G>A	CTA GTT TCA AAG ACC CGA AGA TTA G
LCP1	1689C>A	AAC CAG GTT CAA TTA ACT ATG ACC
LCP1	1803A>T	ATG CCC TGC CTG AAG ACC TGG
LCP1	1857C>G	TTG CCT GCC TGA TGG GGA AAG
LCP1	192C>Tr	CTG TAG CCA TCA GGT TTT C
LCP1	204G>Ar	GGT CCA GAT CAC CTG TAG
LCP1	339C>Ar	ACT GCT CTG AAG TAC CAC
LCP1	357A>Gr	GTT GGG TGC CAA CGC TAG
LCP1	423G>Ar	GAT CAT TTT CCA GGG CTT TG
LCP1	480C>Tr	CAG CAT TAA AGA GAT CAT TCG
LCP1	765T>Gr	AAA GAG CAA TCA GAG CTT CAT TTC
LCP1	1026G>Ar	GCA GCA TGC ATT CTG CCC TCT GG
LCP1	1224T>Cr	TCA TCC AGT TCC TAA ATG TCC
LCP1	1335G>Ar	CTC TGT TCC AGT CAA CAG
LCP1	1389G>Ar	AAT TCT CAA GCT TCT TCA TAT TG
LCP1	1404G>Ar	CTA CCG CGT AGT TAC AAT TC
LCP1	1413G>Ar	TCC CCA ATT CTA CCG CGT
LCP1	1492C>Tr	AAG GCC AGT GTG AGA GTG
LCP1	1518C>Tr	TAT ACC TTC TCA TTA GCT GC
LCP1	1539C>Tr	TTT CTT CGA GGA TAT TCA GTG TAT AC
LCP1	1575G>Ar	CAA TAA TGT CAT CAT TGA CCT TC
LCP1	1626G>Ar	AGA TGG ATG AAC TTT TCT TTG C
LCP1	1689C>Ar	GGA TGG CAT CGA TGA GGT C

LCP1	1803A>Tr	ACA CTC TTG CTC CAA TTT TTC G
LCP1	1857C>Gr	ACA CGG TCA TGA CCA TTT TG
LCP1	T7	TAATACGACTCACTATAGGGTAGTCACTTCCTGCCTTG
LCP1	T7r1	GAGCATCATTCTCTTTTCATT
LCP1	T7r2	GGCAGTAACATTCTTTCTTCCTGG
TPT1	T7	TAA TAC GAC TCA CTA TAG GGC CCC TCC CCC CGA GCG CCG
TPT1	T7r	GCA TTC TCT CAA ATG AGT TTA AAT GC
TPT1	T7r2	CCC TGC AGT TCA GAA TGA CAG
TPT1	COSMIC_c.210C>T	TCA CTG GTG TTG ATA TTG TCA TG
TPT1	COSMIC_c.210C>Tr	TTA CTG TGC TTT CGG TAC
TPT1	COSMIC_c.360A>G	GGG CTG CAG AGC AAA TCA AGC
TPT1	COSMIC_c.360A>Gr	CTG TCA TAA AAG GTT TTA CTC TTT C
TPT1	5utr_208	CTC CCT TCA GCC GCC ATC ATG ATT ATC TAC
TPT1	5utr_208r	ACG ACG ACG GCG CTA GCT
TPT1	5utr_192	CTA GCG CCG TTG TCG TCT CCC
TPT1	5utr_192r	CTT AGC ACG AGC CTG AAA CTC
TPT1	5utr_191	GCT AGC GCC GAC GTC GTC TCC
TPT1	5utr_191r	TTA GCA CGA GCC TGA AAC TCG
TPT1	5utr_187	CTA AGC TAG CAC CGT CGT CGT
TPT1	5utr_187r	CAC GAG CCT GAA ACT CGG
TPT1	5utr_183	CGT GCT AAG CCA GCG CCG TCG
TPT1	5utr_183r	AGC CTG AAA CTC GGA GCG
TPT1	5utr_174	TTT CAG GCT CCT GCT AAG CTA GCG CCG
TPT1	5utr_174r	CTC GGA GCG AGC GCG GTG
TPT1	5utr_171	GAG TTT CAG GTT CGT GCT AAG CTA GCG
TPT1	5utr_171r	GGA GCG AGC GCG GTG CAG
TPT1	5utr_167	CTC CGA GTT TTA GGC TCG TGC TAA GCT AGC
TPT1	5utr_167r	CGA GCG CGG TGC AGC CGG
TPT1	5utr_165	CGC TCC GAG TGT CAG GCT CGT GC
TPT1	5utr_165r	AGC GCG GTG CAG CCG GAG
TPT1	5utr_132	TCC CCC CGA GTG CCG CTC CGG
TPT1	5utr_132r	GGG GGG AGC GGG CGG AAA AG
TPT1	5utr_128	CCC CTC CCC CTG AGC GCC GCT
TPT1	5utr_128r	GGA GCG GGC GGA AAA GGC CG
TPT1	5utr_125	TCC CCC CTC CGC CCG AGC GCC
TPT1	5utr_125r	GCG GGC GGA AAA GGC CGA CTC
TPT1	5utr_123	GCT CCC CCC TGC CCC CGA GCG
TPT1	5utr_123r	GGG CGG AAA AGG CCG ACT CAG C
TPT1	3utr_934	ATA AAA TGC AGT TAA ACT CAT TTG AG
TPT1	3utr_934r	TTT TAG ACA ACC TAC ATG AC
TPT1	3utr_867	TGA CTG TGA TAT ATT TGG AGT GG
TPT1	3utr_867r	AAA TAA ATG AAG AGC TCA AGA TG

TPT1	3utr_861	TTA TTT TGA CCG TGA TTT ATT TGG AGT G
TPT1	3utr_861r	ATG AAG AGC TCA AGA TGA C
TPT1	3utr_860	TTT ATT TTG ATT GTG ATT TAT TTG GAG TG
TPT1	3utr_860r	TGA AGA GCT CAA GAT GAC
TPT1	3utr_859	ATT TAT TTT GGC TGT GAT TTA TTT GG
TPT1	3utr_859r	GAA GAG CTC AAG ATG ACA TC
TPT1	3utr_850	GAG CTC TTC AGT TAT TTT GAC TG
TPT1	3utr_850r	AAG ATG ACA TCA GTC CCA TTT G
TPT1	3utr_817	AGG ACT TAA GGC AAA TGG GAC
TPT1	3utr_817r	GGT GTT GTG TGG ATG ACA AG
TPT1	3utr_740	GTT AAC AAA TAT GGC AAT TAT TTT GG
TPT1	3utr_740r	ATT TTT CCA TTT CTA AAC CAT CC
